# Supplementary material for: Blaming luck, claiming skill: Self-attribution bias in error assignment
Source: PLoS Comput Biol. 2025 Dec 16;21(12):e1013787. doi: 10.1371/journal.pcbi.1013787 (PMC12753049; doi:10.1371/journal.pcbi.1013787)
Supplement: S1 Text — Fig A. Histogram of durations, measured in number of trials, of hidden task state sequences (mean = 28.8, standard deviation = 1.5). Each colour represents one participant (N = 51). Fig B. The task was calibrated such that participants would obtain roughly 50% positive scores in both skill and random states. Note that, nevertheless, the overall ratio of positive scores was significantly larger in the skill state compared to the random state at the group level (Wilcoxon signed-rank test, Z = 2.58, p = 0.01). Fig C. (1) Distribution of log-transformed reaction times in rule inference, divided by score (positive, negative). (2) Distribution of hit locations, expressed in pixels as the distance from the mole centre and divided by score (positive, negative). Fig D. Participants’ hit location patterns are expressed as the distance of the hit from the mole centre. (1) distance from the centre divided by hidden task state (random, skill). (2) distance from the centre around task state switches, plotted from -4 trials before the switch to +8 trials after the switch. The trajectories are plotted separately for the two types of transition, from random to skill and skill to random. (3) cross-trial dynamics of participants’ hit behaviour, separately for trials in which participants chose random or skill after obtaining a negative or positive score. The plot shows that participants adjusted their hit locations accordingly. After receiving a negative score, participants tended to hit closer to the centre (increased precision of hits). In contrast, after receiving a positive score, participants tended to hit further away from the centre (a relaxation of the precision). Fig E. Comparison of the full model against alternative models, in which specific parameters, or sets thereof, were fixed (e.g., in which the error sensitivity α was constrained to be score-independent). We used the Akaike Information Criterion (AIC) [59,60] to compare models. Note that lower AIC values mean better m [file pcbi.1013787.s001.pdf]

Supplementary Information file

Blaming Luck, Claiming Skill: Self-Attribution Bias in Error Assignment

Naoyuki Okamoto[1,2], Michael Taylor[3], Takatomi Kubo[1,4], Shin Ishii[2,5,6], Benedetto De Martino[1,3,*,$] & Aurelio Cortese[1,7,8,*,$]

[1] Computational Neuroscience Laboratories, ATR Institute International, 619-0288 Kyoto, Japan
[2] Graduate School of Informatics, Kyoto University, 606-8501 Kyoto, Japan
[3] Institute of Cognitive Neuroscience, University College London, WC1N 3AZ, London, UK
[4] Graduate School of Information Science, Nara Institute of Science & Technology, 630-0192 Nara, Japan
[5] Neural Information Analysis Laboratories, ATR Institute International, 619-0288 Kyoto, Japan
[6] International Research Center for Neurointelligence (WPI-IRCN), Institutes for Advanced Study, University of Tokyo, 113-0033 Tokyo, Japan
[7] Department of Biomedical Engineering, Sungkyunkwan University, Suwon, South Korea
[8] Center for Neuroscience Imaging Research, Institute for Basic Science, Suwon, South Korea

[$] Equally contributor authors
* benedettodemartino@gmail.com, cortese.aurelio@gmail.com

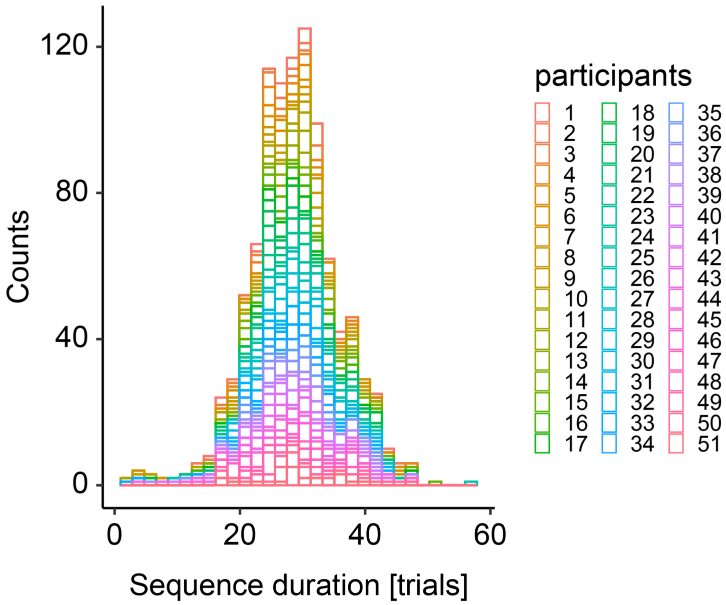

Fig A. Histogram of durations, measured in number of trials, of hidden task state sequences (mean = 28.8, standard deviation = 1.5). Each colour represents one participant (N = 51).

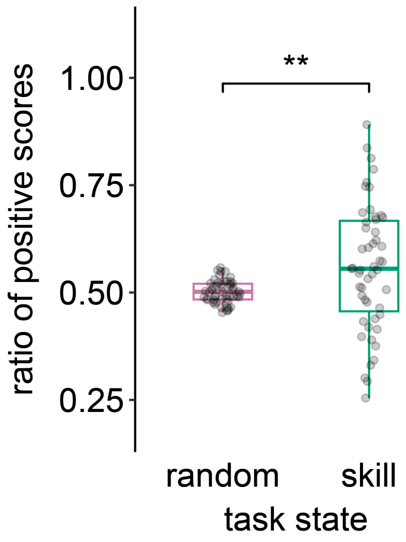

Fig B. The task was calibrated such that participants would obtain roughly 50% positive scores in both skill and random states. Note that, nevertheless, the overall ratio of positive scores was significantly larger in the skill state compared to the random state at the group level (Wilcoxon signed-rank test, Z = 2.58, p = 0.01).

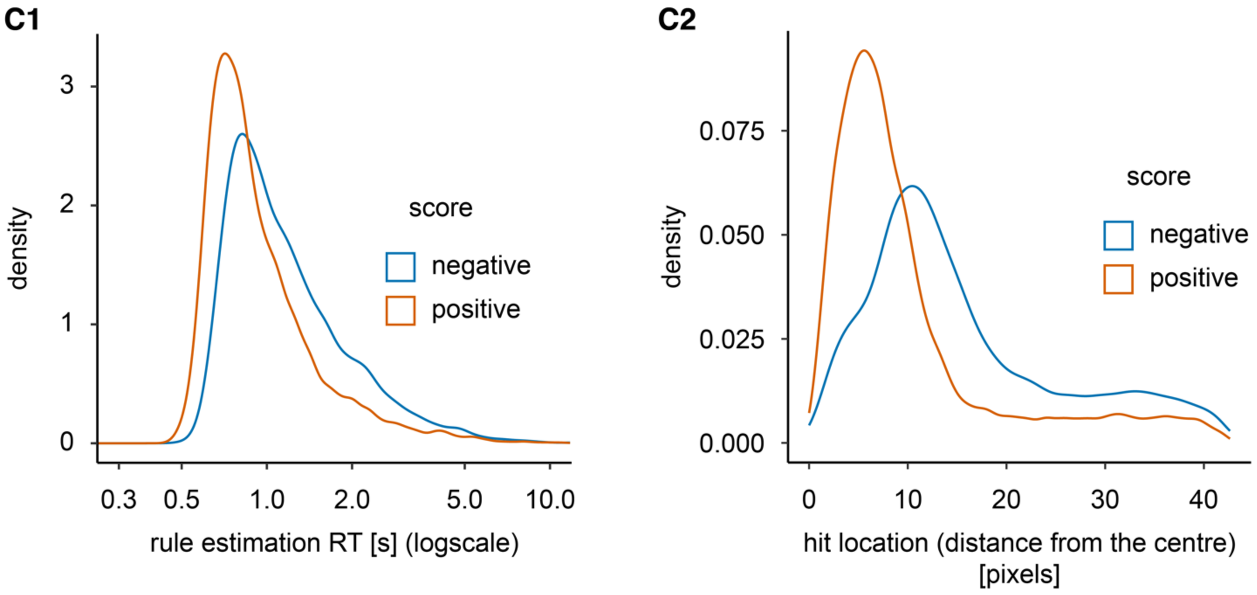

Fig C. (C1) Distribution of log-transformed reaction times in rule inference, divided by score (positive, negative). (C2) Distribution of hit locations, expressed in pixels as the distance from the mole centre and divided by score (positive, negative).

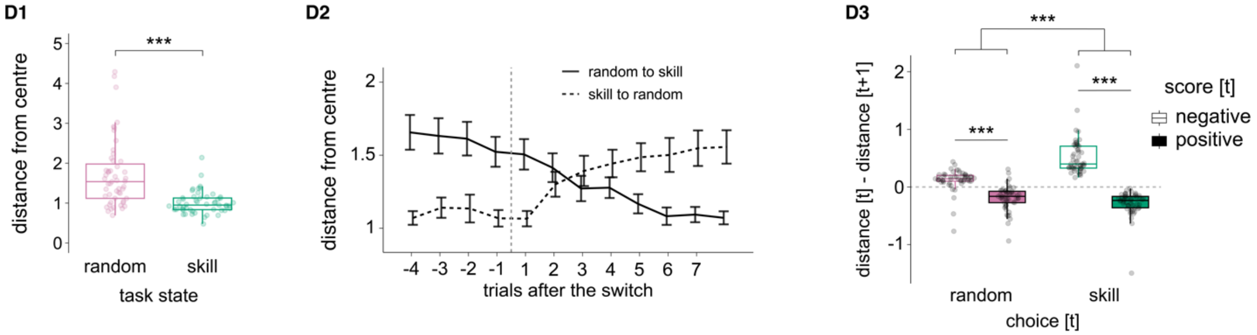

Fig D. Participants' hit location patterns are expressed as the distance of the hit from the mole centre. (D1) distance from the centre divided by hidden task state (random, skill). (D2) distance from the centre around task state switches, plotted from -4 trials before the switch to +8 trials after the switch. The trajectories are plotted separately for the two types of transition, from random to skill and skill to random. (D3) cross-trial dynamics of participants' hit behaviour, separately for trials in which participants chose random or skill after obtaining a negative or positive score. The plot shows that participants adjusted their hit locations accordingly. After receiving a negative score, participants tended to hit closer to the centre (increased precision of hits). In contrast, after receiving a positive score, participants tended to hit further away from the centre (a relaxation of the precision).

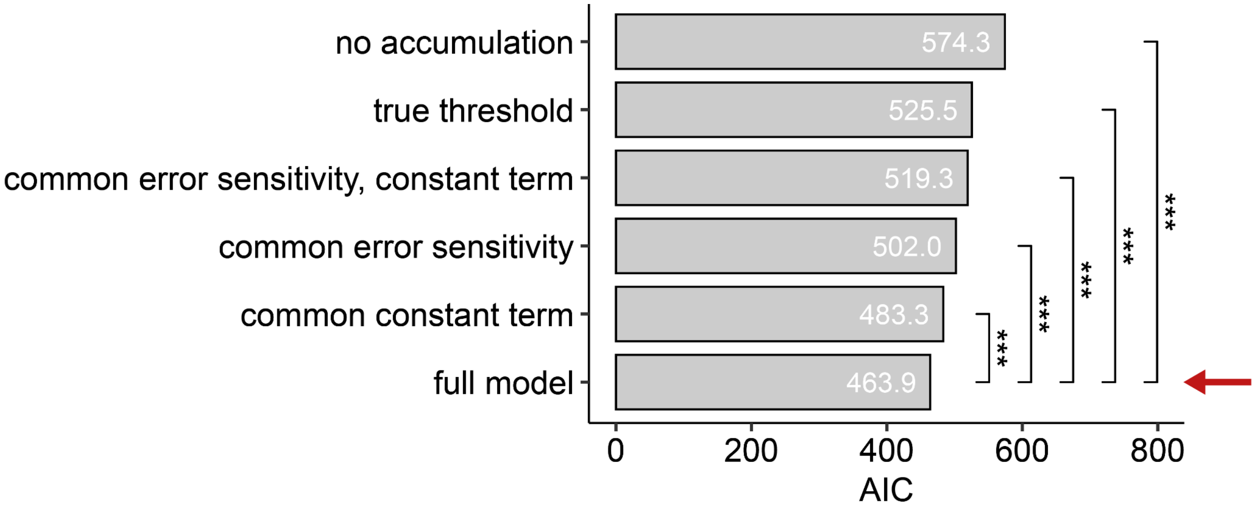

Fig E. Comparison of the full model against alternative models, in which specific parameters, or sets thereof, were fixed (e.g., in which the error sensitivity α was constrained to be score-independent). We used the Akaike Information Criterion (AIC) (Akaike 1974; Symonds and Moussalli 2010) to compare models. Note that lower AIC values mean better model fit. The plot shows each model's average AIC values (computed across participants). Wilcoxon signed-rank test, with FDR correction for multiple comparisons, demonstrated that the full model provided a significantly better fit for participants' data. It also showed that the subjective threshold parameter played a more important role than the constant term or the error sensitivity (both reflecting a positivity bias) due to the larger difference in AIC compared to the full model. The red arrow indicates the best-fit model, *** $P < 0.001$.

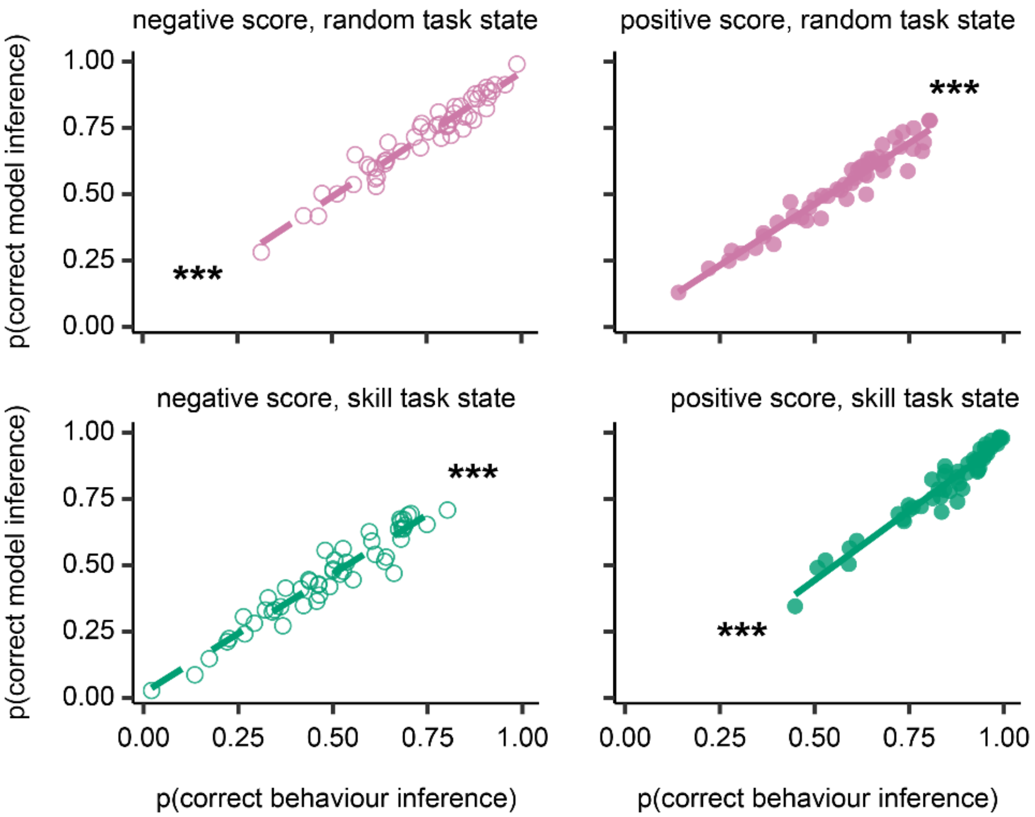

Fig F. Quantitative correspondence between participants' behaviour and the model's simulated behaviour in terms of p(correct state inference). Each subplot represents one of the four conditions based on scores (negative, positive) and hidden task states ('random', 'skill'). From top left to bottom right: negative score, random task state (r = 0.96, $P < 10^{-10}$); positive score, random task state (r = 0.96, $P < 10^{-10}$); negative score, skill task state (r = 0.96, $P < 10^{-10}$); positive score, skill task state (r = 0.96, $P < 10^{-10}$). Each plot N = 51, circles represent individual participants, and solid/dotted lines represent the linear fit.

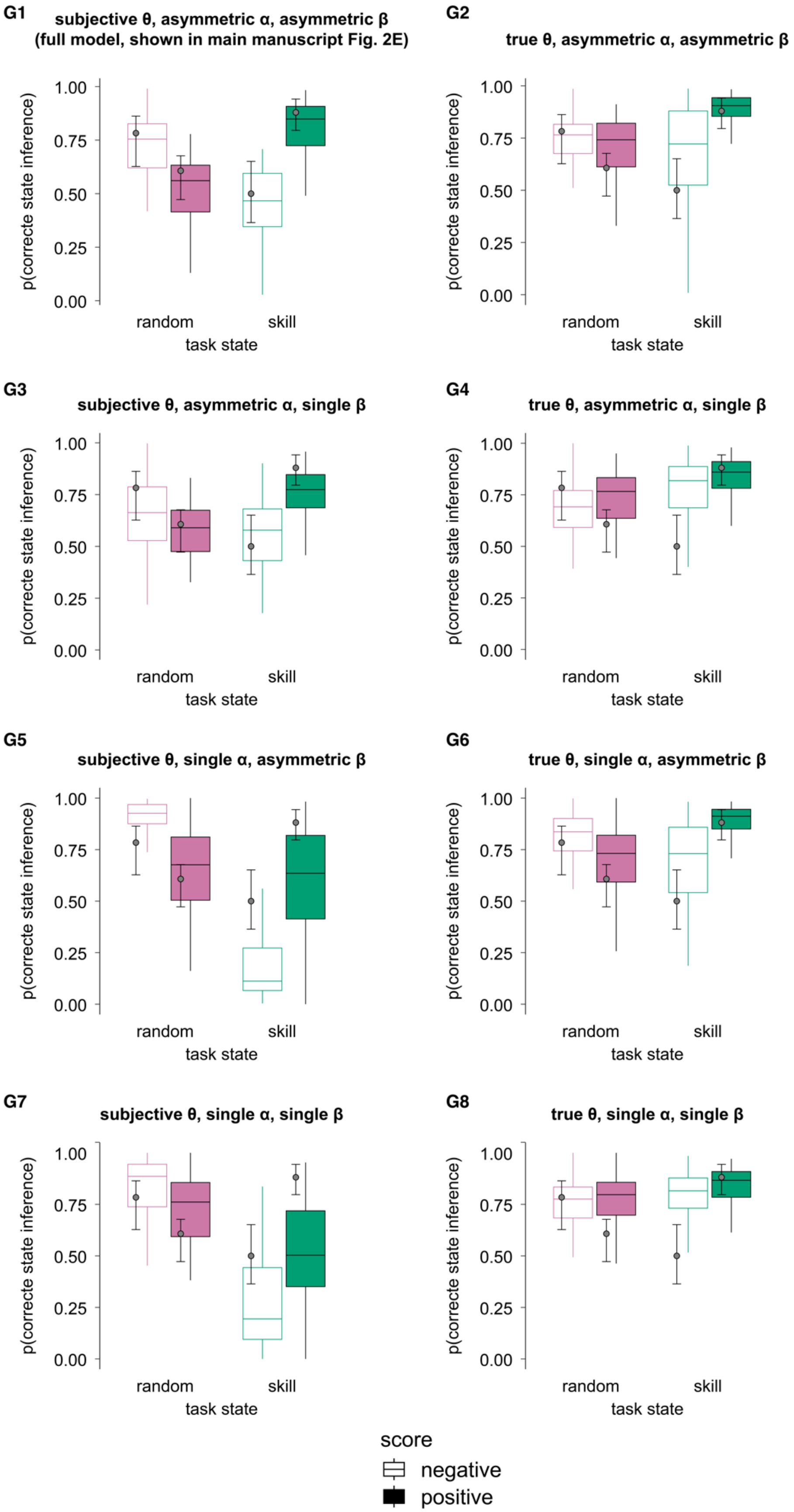
G1
subjective θ, asymmetric α, asymmetric β
(full model, shown in main manuscript Fig. 2E)
G2
true θ, asymmetric α, asymmetric β
G3
subjective θ, asymmetric α, single β
G4
true θ, asymmetric α, single β
G5
subjective θ, single α, asymmetric β
G6
true θ, single α, asymmetric β
G7
subjective θ, single α, single β
G8
true θ, single α, single β
p(correcte state inference)
1.00
0.75
0.50
0.25
0.00
random
skill
task state
score
negative
positive

Fig G. Inference accuracy, plotted by hidden task state and score. Simulations with specific parameter settings to evaluate the effect of cognitive strategies on inference accuracy. We performed eight simulations based on three parameters, with two possible settings each. Across plots, the small circles represent participants' average inference accuracy, and the error bars are the standard error of the mean. The boxplots represent the simulation median and interquartile ranges. (G1) Simulating the best fitting model with subjective threshold, asymmetric (score-dependent) error sensitivity, and asymmetric (score-dependent) constant term. This simulation is the same as reported in the main manuscript in Fig 2E. Note the close mapping between participants' behaviour and simulation results. (G1, G3, G5, G7) Simulations based on the model with the subjective threshold, modifying the error sensitivity and/or the constant term. (G2, G4, G6, G8) Simulations based on the model with the true threshold, modifying the error sensitivity and/or constant term. (G8) Oracle agents use the true threshold and equal weights across positive and negative scores for error sensitivity and constant term. For all panels, simulation results are plotted as boxplots, with the box representing the median, first and third quartiles and whiskers representing the minimum and maximum of the data range, while participants results are plotted as circles representing the median and error bars representing the first and third quartiles.

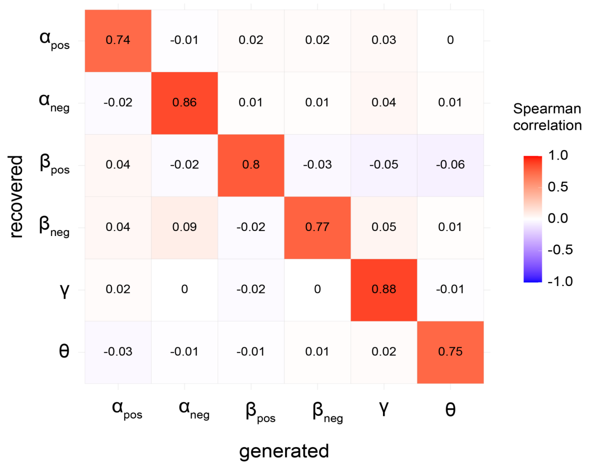

Fig H. Parameter recovery analysis, showing the confusion matrix with correlations between original (generated) parameters and recovered parameters.

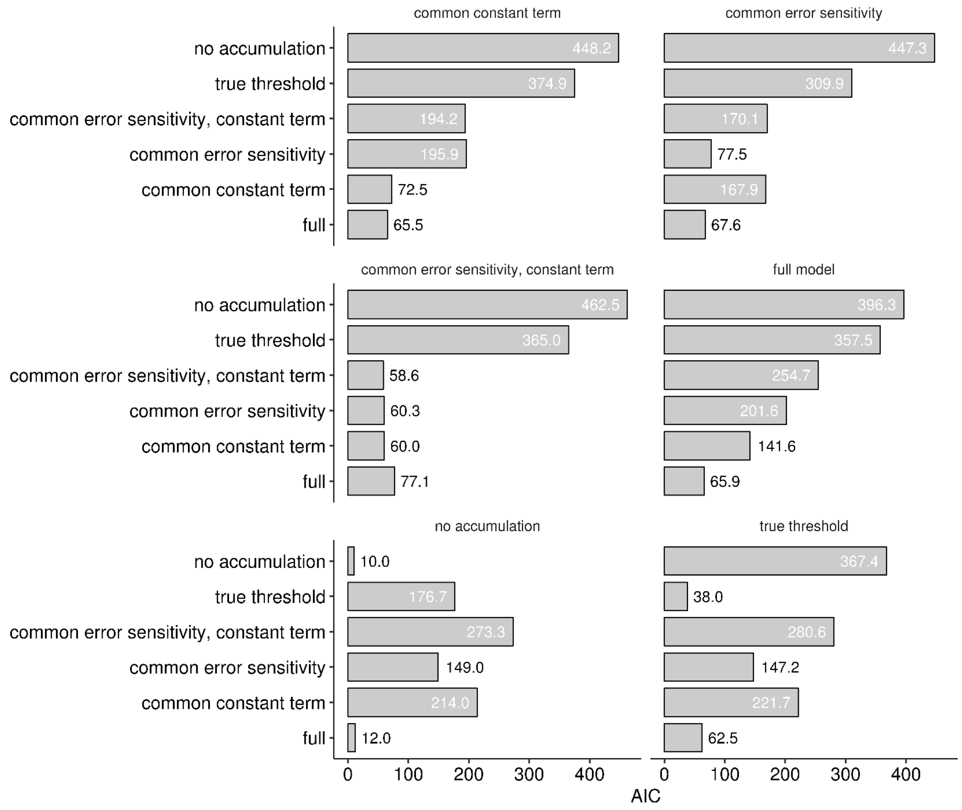

Fig I. Model recovery analysis. We first simulated data with each model using parameters within the range of fitted values from our participants' data, then fit each model to simulated data. All model comparisons displayed here were done using AIC. For all cases, the original ground truth model was consistently ranked as best fitting, in addition to the full model, since all alternative models are nested versions of the full model in which a parameter has been set to a fixed value.

J1 **subjective θ, asymmetric α, asymmetric β (full model, shown in main manuscript Fig. 2E)**

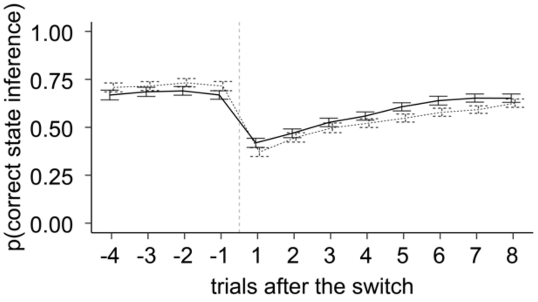

J2 **true θ, asymmetric α, asymmetric β**

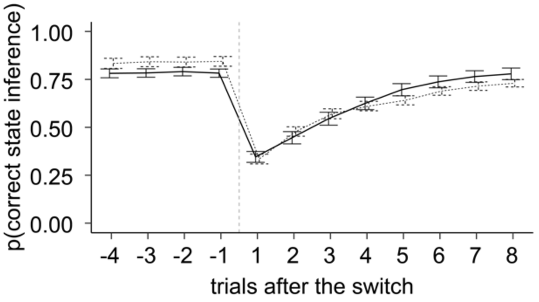

J3 **subjective θ, asymmetric α, single β**

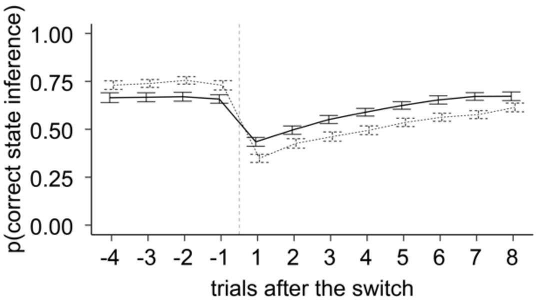

J4 **true θ, asymmetric α, single β**

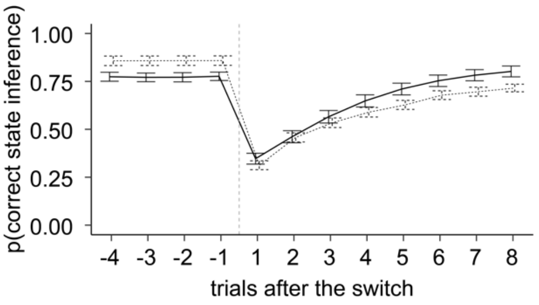

J5 **subjective θ, single α, asymmetric β**

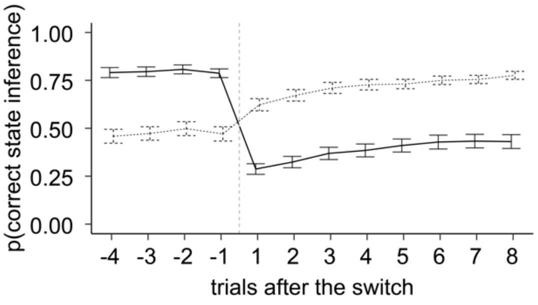

J6 **true θ, single α, asymmetric β**

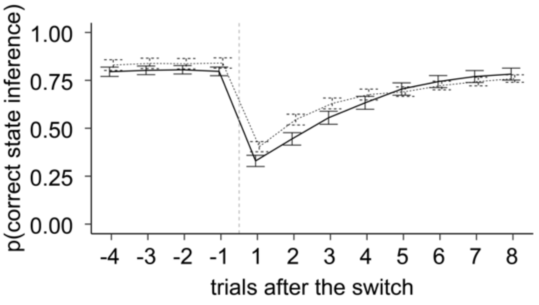

J7 **subjective θ, single α, single β**

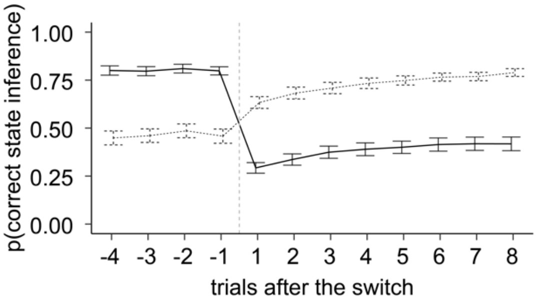

J8 **true θ, single α, single β**

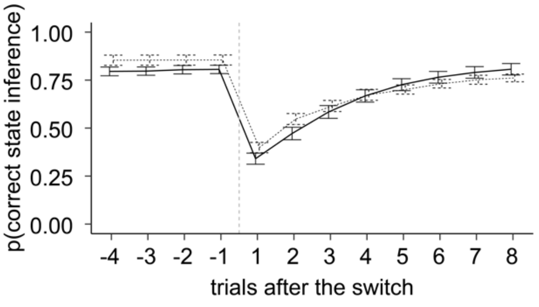

Fig J. Inference accuracy, plotted around hidden task state switches. Simulations with specific parameter settings to evaluate the effect of cognitive strategies on inference accuracy. We performed eight simulations based on three parameters, with two possible settings each. Across plots, the small circles represent participants' average inference accuracy, and the error bars represent the standard error of the mean. The boxplots represent the simulation median and interquartile ranges. (J1) Simulating the best fitting model with subjective threshold, asymmetric (score-dependent) error sensitivity, and asymmetric (score-dependent) constant term. This simulation is the same as reported in the main manuscript in Fig 2E. Note the close mapping between participants' behaviour and simulation results. (J1, J3, J5, J7) Simulations based on the model with the subjective threshold, modifying the error sensitivity and/or the constant term. (J2, J4, J6, J8) Simulations based on the model with the true threshold, modifying the error sensitivity and/or constant term. (J8) Oracle agents use the true threshold and equal weights across positive and negative scores for error sensitivity and constant term.

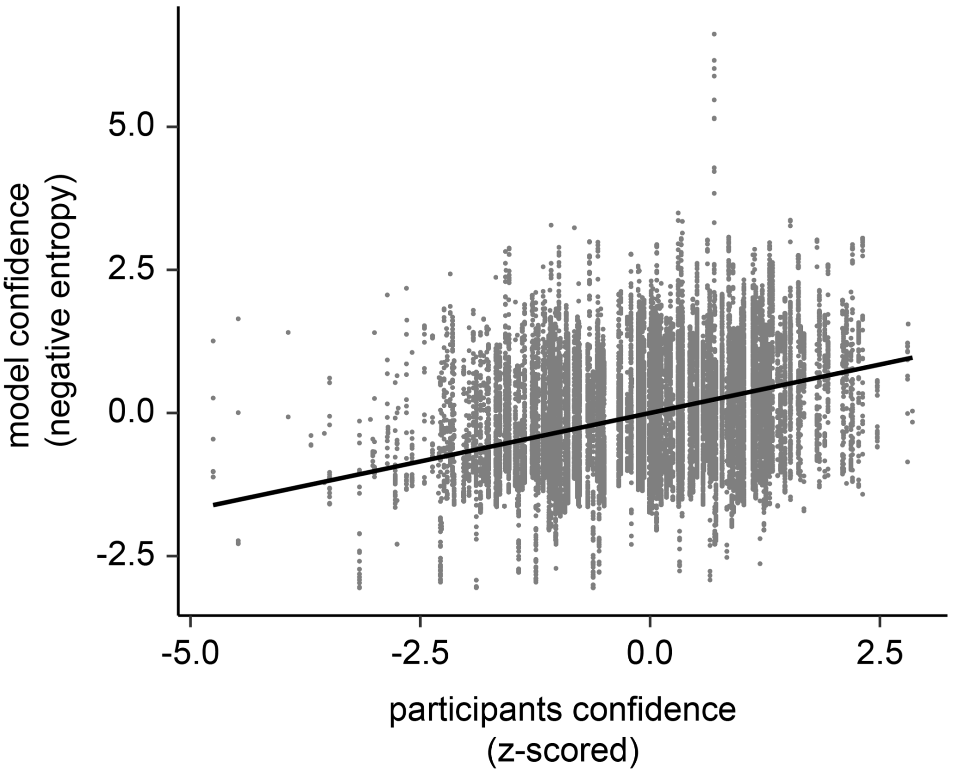

Fig K. Correspondence between participants' confidence judgements and negative entropy of the model's decision output. Importantly, the model was not optimised based on confidence but solely based on the inference choices. The model's confidence was taken simply as the negative entropy of the decision output (since entropy signals the uncertainty in that decision). We evaluated the linear relationship with a linear mixed-effect model [Wilkinson formula *negative_entropy ~ confidence + (confidence | subjID)*]. The factor confidence was significant (estimate = 0.34, std = 0.026, t_{50} = 12.86, P < 0.001).

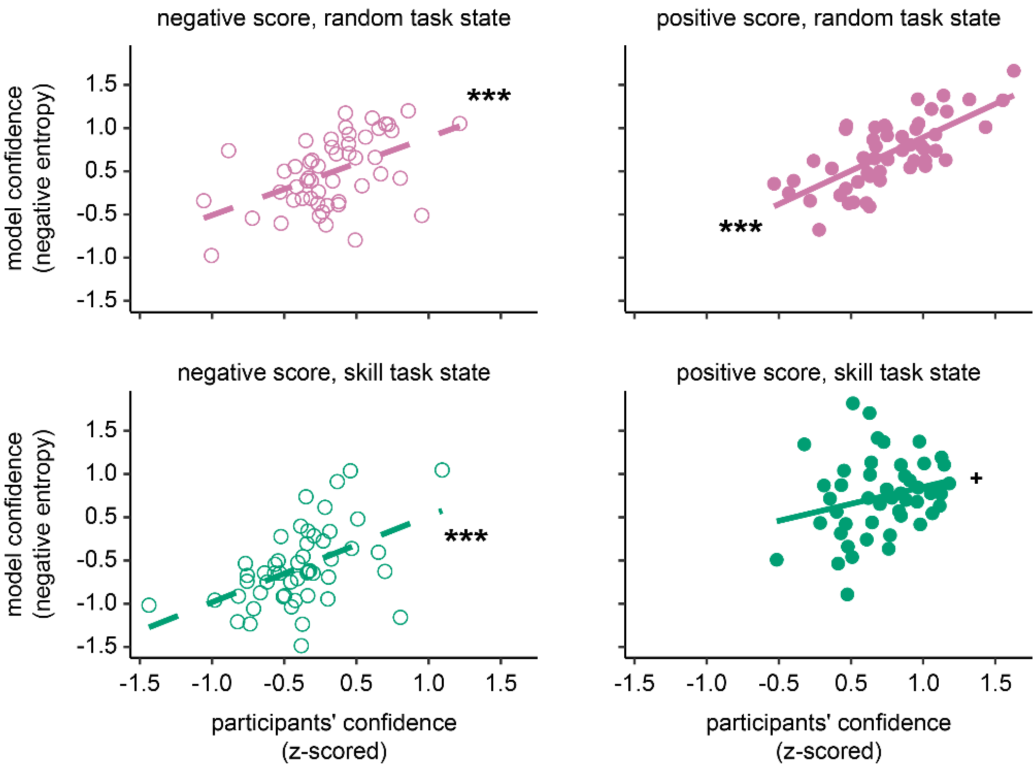

Fig L. Quantitative correspondence between participants' behaviour and the model's simulated behaviour in terms of p(correct state inference). Each subplot represents one of the four conditions based on scores (negative, positive) and hidden task states ('random', 'skill'). From top left to bottom right: negative score, random task state (r = 0.49, $P < 0.001$); positive score, random task state (r = 0.69, $P < 10^{-4}$); negative score, skill task state (r = 0.52, $P < 0.001$); positive score, skill task state (r = 0.26, $P = 0.069$). Each plot N = 51, circles represent individual participants, and solid/dotted lines represent the linear fit.

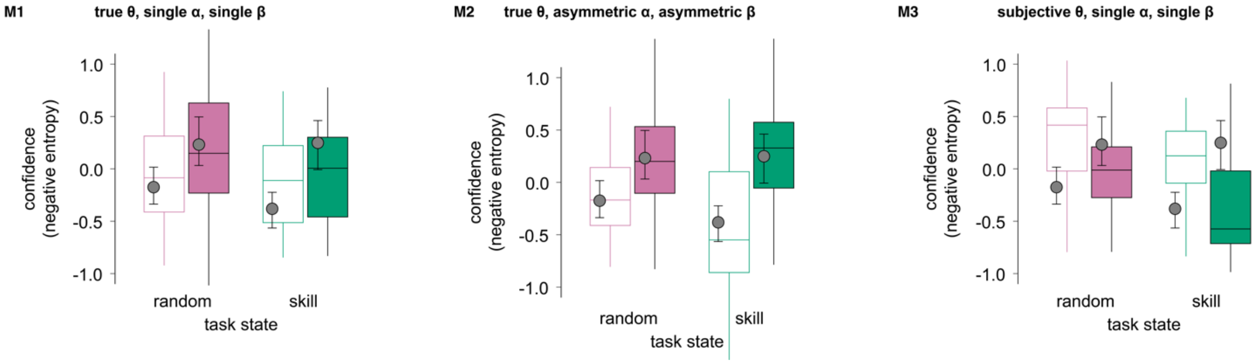

Fig M. Confidence (negative entropy), plotted by hidden task state and score. Simulations with specific parameter settings to evaluate the effect of cognitive strategies on confidence. (M1) Simulating oracle agents, using the true threshold and equal weights across positive and negative scores for error sensitivity and bias. Confidence shows minimal variation across hidden task states and scores. (M2) Simulating agents with the true threshold but with a score-dependent error sensitivity and bias. Confidence again displays the original effect of scores, with higher confidence for positive scores than negative ones. (M3) Simulating agents using the subjective threshold and score-independent error sensitivity and bias. Confidence shows the opposite pattern, being lower for positive scores and higher for negative scores and overall lower in skill compared to random task state. For all panels, simulation results are plotted as boxplots, with the box representing the median, first and third quartiles and whiskers representing the minimum and maximum of the data range, while participants results are plotted as circles representing the median and error bars representing the first and third quartiles.

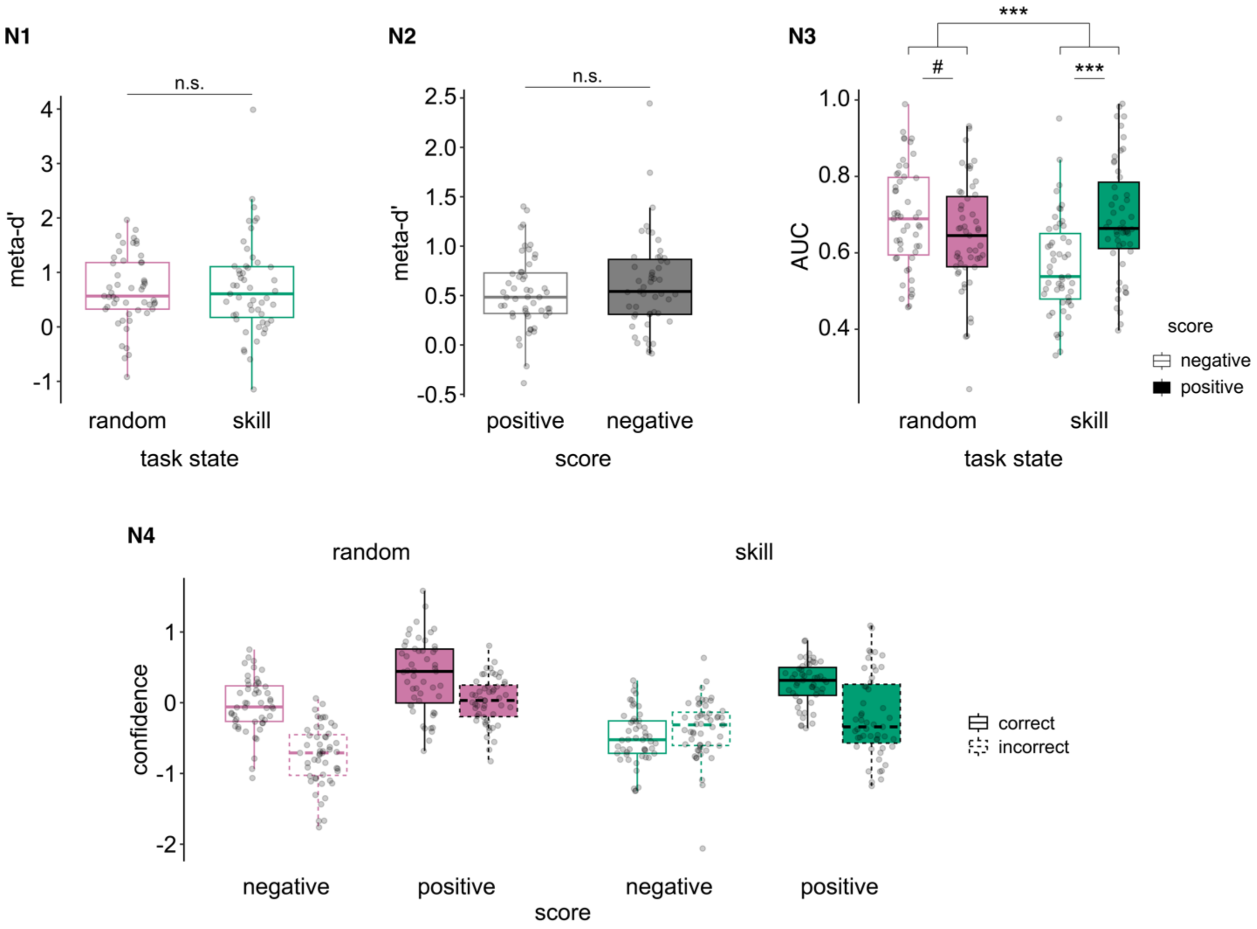

Fig N. Metacognition. (N1) Formal meta-d' analysis for each task state (random and skill). Participants had equal metacognitive capacity across states. HMeta-d was used to calculate meta-d' (Fleming 2017). (N2) Formal meta-d' analysis based on the score obtained (negative, positive). Participants had equal metacognitive capacity across score types. (N3) Area under the curve (AUC) analysis separately for each score-task state combination. Note that because we are now looking at each condition separately, it is no longer possible to compute meta-d' (which requires data from both responses within each condition of interest). We thus computed AUC on the confidence-choice response operating curve. Note the steep drop in AUC (metacognitive ability) specific to the negative scores in the skill task state. (N4) Showing the data from panel N3, further separated for correct and error trials. Notice the absence of a difference in confidence between correct and error trials, specifically after the negative scores in the skill task state. For all boxplots, the box represents the median, first and third quartiles, and whiskers represent the minimum and maximum of the data range; scatter plots represent participants' individual data points.

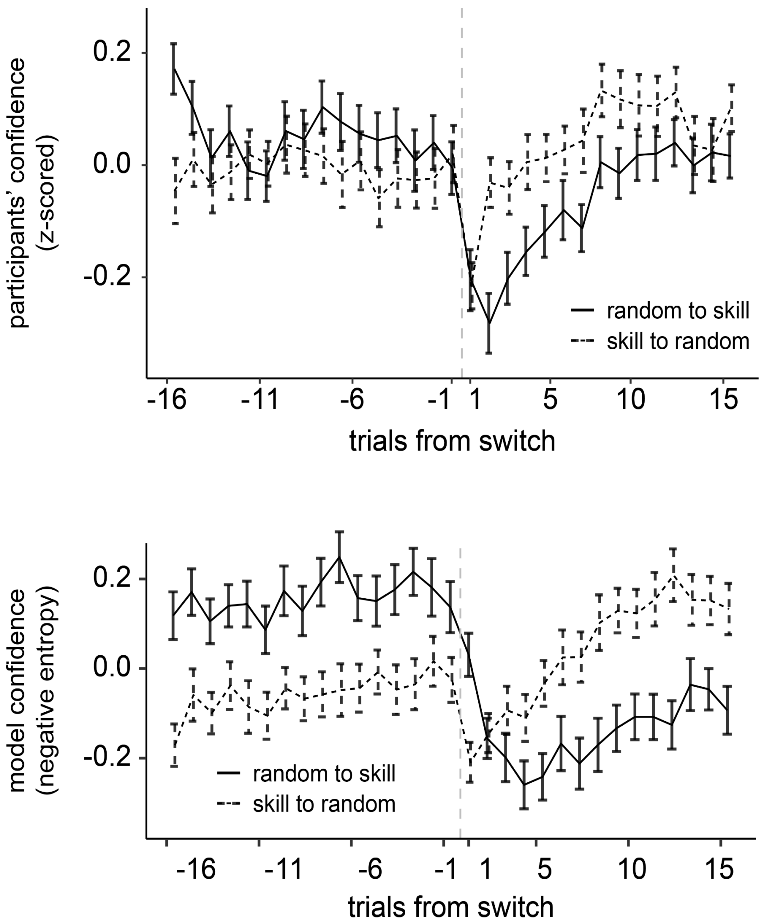

Fig O. Time series of participants' confidence judgements (top) and model confidence (bottom) around hidden task state switches (e.g., random → skill or skill → random). The central line represents the mean across participants, and the error bars represent the standard error of the mean. Confidence was first z-scored and averaged within each participant/simulation. In both cases, the random condition leads to a higher average confidence than the skill condition. While this effect was stronger in the model results, it was also clearly present in participants' data.

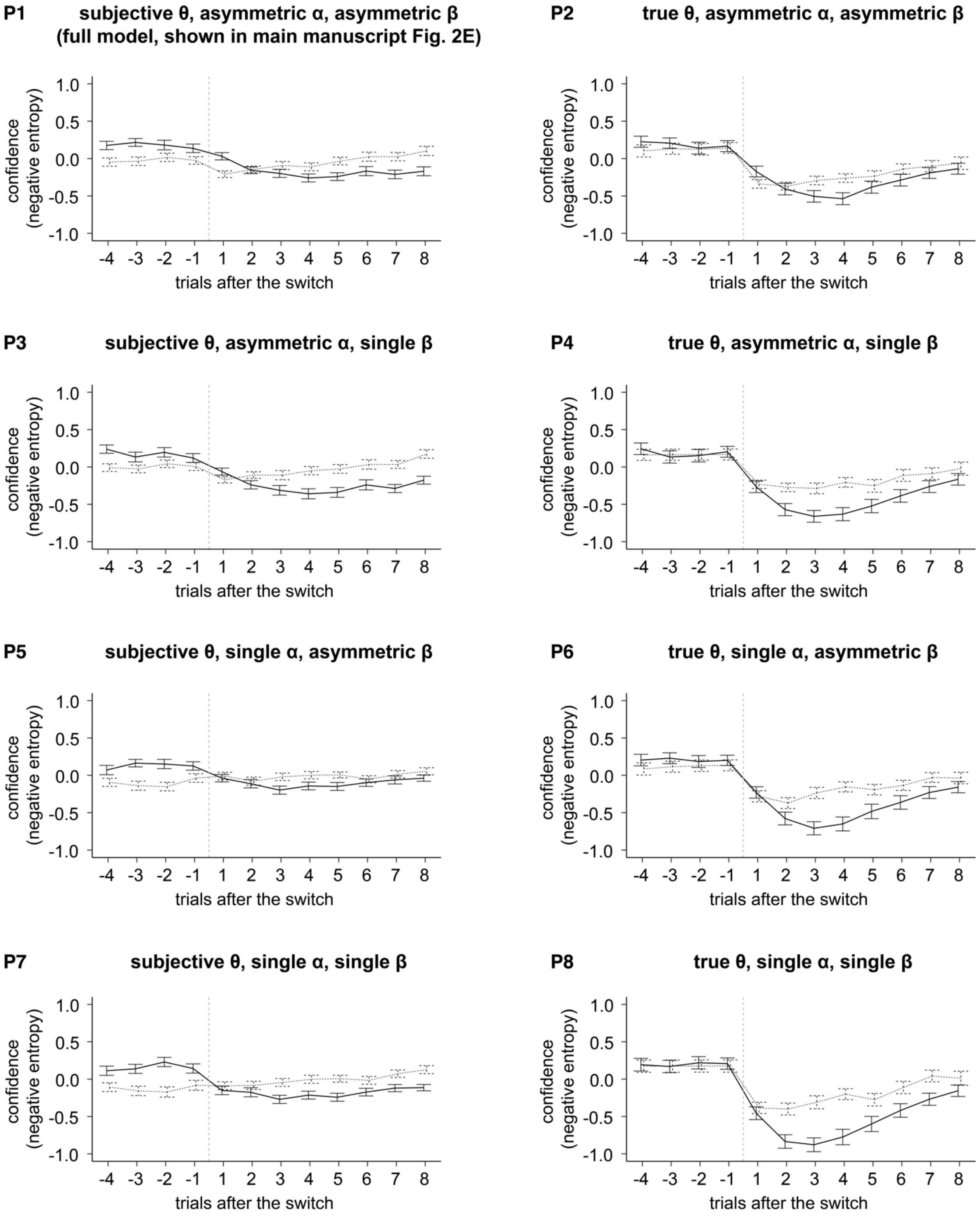

Fig P. Confidence (negative entropy), plotted around hidden task state switches. Simulations with specific parameter settings to evaluate the effect of cognitive strategies on inference accuracy. We performed eight simulations based on three parameters, with two possible settings each.

Across plots, the small circles represent participants' average inference accuracy, and the error bars are the standard error of the mean. The boxplots represent the simulation median and interquartile ranges. (P1) Simulating the best fitting model with subjective threshold, asymmetric (score-dependent) error sensitivity, and asymmetric (score-dependent) constant term. This simulation is the same as reported in the main manuscript in Fig 2E. Note the close mapping between participants' behaviour and simulation results. (P1, P3, P5, P7) Simulations based on the model with subjective threshold, modifying the error sensitivity and/or the constant term. (P2, P4, P6, P8) Simulations based on the model with the true threshold, modifying the error sensitivity and/or constant term. (P8) Oracle agents use the true threshold and equal weights across positive and negative scores for error sensitivity and constant term.

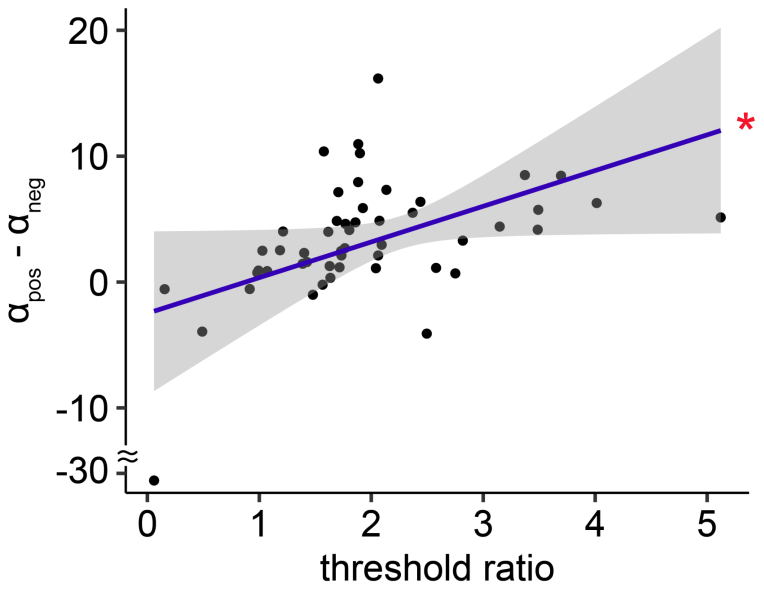

Fig Q. The plot shows the linear relationship between the perceptual distortion (represented as the ratio between the subjective and true thresholds) and the strength of the positivity/confirmation bias (defined as the difference between the error sensitivity for positive vs negative outcomes). We used robust regression to evaluate the strength of the relationship (slope = 2.84 ± 1.40, t_{49} = 2.02, P = 0.049). Dots represent individual participants, the line represents the linear fit, and the red asterisk indicates P < 0.05.

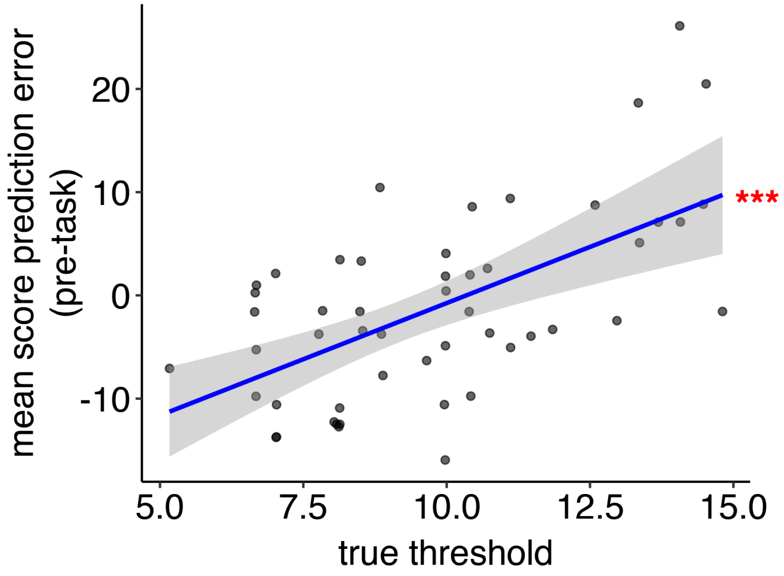

Fig R. The plot shows the linear relationship between the individually calibrated true threshold computed as the median of all hit locations during the score prediction task, and the average signed score prediction error. A larger threshold means participants hit, on average, further from the centre of the mole, and a positive error means participants overestimated their ability to hit the centre. Thus, the x-axis represents motor ability, while the y-axis represents bias in the subjective evaluation of motor ability. We used robust regression to evaluate the strength of the relationship (slope = 2.18, t_{49} = 4.58, $p < 0.001$). Dots represent individual participants, the line represents the linear fit, and the red asterisk indicates $P < 0.001$.

		task state	
		skill	random
choice	skill	0.70 ± 0.13	0.35 ± 0.13
	random	0.30 ± 0.13	0.65 ± 0.13

Table A. Confusion matrix of task state and choices. Each cell is the ratio of choices within the relevant task state. The ratios within each task state sum to one within participants and are reported as mean ± STD computed across participants. Wilcoxon signed rank test on diagonal elements, i.e., true positive and true negative rates: $Z = 1.87$, $P = 0.061$.

Model	Free parameters	Mean AIC / BIC / LL
**Full model**	$\boldsymbol{\alpha_{pos}, \alpha_{neg}, \beta_{pos}, \beta_{neg}, \gamma, \theta}$	**463.9 / 489.9 / -226.0**
Single constant term	$\alpha_{pos}, \alpha_{neg}, \beta, \gamma, \theta$	483.3 / 504.9 / -236.7
Single error sensitivity	$\alpha, \beta_{pos}, \beta_{neg}, \gamma, \theta$	502.0 / 523.7 / -246.0
Single error sensitivity, constant	$\alpha, \beta, \gamma, \theta$	519.3 / 536.6 / -255.7
Fixed, true threshold	$\alpha_{pos}, \alpha_{neg}, \beta_{pos}, \beta_{neg}, \gamma$	525.5 / 547.1 / -257.7
No retention factor	$\alpha_{pos}, \alpha_{neg}, \beta_{pos}, \beta_{neg}, \theta$	574.3 / 595.9 / -282.1

Table B. List of models used in the computational analysis of behaviour. The full model, highlighted in bold, provided the best fit across participants (lowest AIC, BIC, LL).

Text A

Task instructions – score prediction

In this pre-task, participants subjectively evaluated their own performance (hit accuracy).

First, the practice session for score prediction was explained. Participants were instructed to hit moles that appeared at random locations on the screen as quickly and accurately as possible with their right index finger. It was explained that after each hit, they would receive feedback on their hit in two forms. A score shown at the bottom of the screen, from 0 to 100, reflecting how close to the centre they hit (0 being the edge, and 100 the centre); a red cross at the hit location; and five concentric circles indicating a set of five distances from the centre. This was done to help participants learn the relationship between where they hit and the score they received.

In the main session, participants were instructed to hit the moles as they did in practice. However, after each hit, they were asked to report the score they believed they had obtained using a slider ranging from 0 to 100. Following that, they were asked to rate their confidence in the accuracy of their score prediction on a 4-point Likert scale (1: low confidence to 4: high confidence), and were encouraged to use the full range of the scale. It was communicated that the correct score would not be displayed after their response in this task.

Task instructions – rule inference

For this task, it was explained that the feedback for hitting a mole would change from a score to binary feedback of "Good" or "Bad". Participants were informed that this feedback was determined based on one of two rules:

1. **Skill Rule:** The evaluation reflects the participant's hit location. Hitting the centre of the mole accurately would result in “Good” feedback, while missing the centre results in “Bad” feedback.
2. **Random Rule:** The feedback is determined probabilistically, regardless of the hit location.

It was also instructed that these two rules would switch at random times during the task, but that once a rule was set, it tended to persist for a while (i.e., that there wouldn’t be switches every few trials).

Then, the practice session for the rule inference task was explained. After hitting a mole and receiving "Good/Bad" feedback, participants would estimate whether the current rule was "Skill" or "Random" and provide their answer. In the practice session, the correct rule was displayed at the end of each trial, providing participants with the opportunity to learn the characteristics of the task and rule inference.

In the main session, participants would hit moles and infer the rule from the given positive/negative feedback, just as in the practice. Afterwards, they were asked to rate their

confidence in their inference on a 4-point scale. It was emphasised that no feedback on the correctness of their rule inference choice would be provided during the main session.

The method for determining the additional reward was clearly communicated: a reward would be added only when one received a positive feedback AND inferred the correct rule. This motivated participants to aim for both high-precision hits and accurate rule inference.

Finally, it was provided as supplementary information that the two rules would appear with roughly equal frequency throughout the entire task.

Supplementary references

Akaike, H. 1974. “A New Look at the Statistical Model Identification.” *IEEE Transactions on Automatic Control* 19 (6): 716–23.

Fleming, Stephen M. 2017. “HMeta-D: Hierarchical Bayesian Estimation of Metacognitive Efficiency from Confidence Ratings.” *Neuroscience of Consciousness* 2017 (1): nix007.

Symonds, Matthew, and Adnan Moussalli. 2010. “A Brief Guide to Model Selection, Multimodel Inference and Model Averaging in Behavioural Ecology Using Akaike’s Information Criterion.” *Behavioral Ecology and Sociobiology* 65 (1): 13–21.
