# Supplementary material for: Blaming luck, claiming skill: Self-attribution bias in error assignment
Source: PLoS Comput Biol. 2025 Dec 16;21(12):e1013787. doi: 10.1371/journal.pcbi.1013787 (PMC12753049; doi:10.1371/journal.pcbi.1013787)
Supplement: Attachment — Submitted filename: Response to Reviewers.pdf [file pcbi.1013787.s004.pdf]

Reviewer #1: The paper continues a recent trend in tackling a question that is important, but challenging, and about which more research is needed. I appreciate the choice of task and the inclusion of confidence estimates in the measures collected. Overall, I have one major concern: I feel the claim regarding a perceptual bias underlying self-serving attribution, which is repeatedly made, is not adequately supported in the current version of the manuscript. I believe more clarity is needed in the argumentation, especially in describing the modelling and how it supports the claim.

I provide more details below, along with minor comments and suggestions.

We thank the reviewer for assessing our manuscript positively and for providing critical and useful feedback that has improved its clarity.

Substance:

1. I think describing theta as modelling participants' perception of their own motor ability needs some discussion, since the threshold, as it is, can also be interpreted as capturing beliefs about how stringent or lax the task requirements are. Indeed, this is the more natural interpretation, given the way the model is formulated, and especially given the fact that the model assumes that the distance between the target centre and hit location is known to participants (assuming I understood correctly). Teasing apart skill and task difficulty is of course very tricky, but there is a conceptual distinction between motor ability (being able to hit close to the centre) and task requirements/ difficulty (what distance to centre is good enough), as well as between perception of motor ability, i.e., distance from hit location to target centre and inference about task requirements, i.e., the threshold for 'good' performance. I think these points need more in-depth discussion. Given this, I am not convinced that the modelling results really can be interpreted to support the claim, which is repeatedly made, that biases operate (also) at a "basic perceptual level".

We thank the reviewer for their insightful comment and fully agree that the discussion could benefit from greater nuance. We also wish to delineate two points that may not have been sufficiently clear in the original submission.

First, while the model assumes that participants have limited access to the precise hit location, this assumption is restricted to a binary distinction—whether the hit landed within the centre area or outside it. The model does not rely on precise spatial information (i.e., the exact distance from the centre), which participants could not access. However, it does incorporate the idea that participants have an internal representation of the centre area—delimited by the hidden threshold—and are therefore able to form a general sense of whether a hit was relatively close to or far from the centre.

Second, task difficulty was individually calibrated during the initial practice (score prediction) session to account for differences in motor ability. Specifically, we used each participant's median hit location during this session to define their objective threshold—the point that determines feedback by separating centre and edge regions.

Regarding the reviewer's main question—whether the task and modelling results support the claim that attribution biases extend to basic perceptual levels—we highlight two additional findings that support this interpretation.

First, participants who tended to overestimate their performance during the score prediction task (i.e. believing they had hit more accurately than they actually had) also exhibited a larger objective threshold in the main task. This positive correlation between motor ability and perception of ability (see Figure 1, now added as Supplementary Figure 18; robust regression, slope = 2.18, $t_{49} = 4.58$, $p < 0.001$) supports a perceptual basis for the observed biases.

Second, if the subjective threshold parameter were only reflecting abstract beliefs about task demands, we would expect it to be unrelated to confidence in inference. However, we found that participants' confidence closely tracked the location of the subjective threshold: confidence was lowest near the threshold and highest away from it (see Figure 2 below, now added as panel C to Figure 4 in the main manuscript). This pattern suggests that the subjective threshold parameter captures perceptual processes, not only conceptual beliefs.

That said, we appreciate the reviewer's call for caution in interpretation and have revised the manuscript to better reflect this. In particular, we have recast our conclusions in more nuanced terms and explicitly addressed the alternative interpretations raised by the reviewer in the revised discussion.

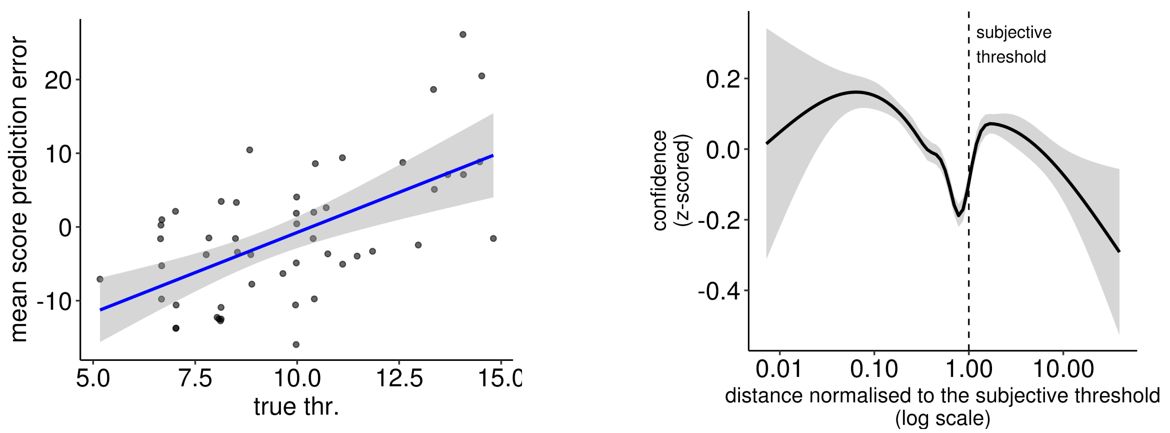

To this end, we have added the following paragraphs in the results, first additional paragraph (p. 6-7, l. 220-226):

Note that while the model assumes that participants have limited access to the precise hit location, this assumption is restricted to a binary distinction—whether the hit landed within the centre area or outside it. The model does not otherwise rely on precise spatial information (i.e., the exact distance from the centre), which participants could not access. However, it does incorporate the idea that participants have an internal representation of the centre area—delimited by the hidden threshold—and are therefore able to form a general sense of whether a hit was relatively close to or far from the centre

In the results, second additional paragraph (p. 11, l. 364-368):

Moreover, we found that confidence accurately reflected task uncertainty with respect to the subjective threshold parameter signalling a shift between positive and negative feedback in the skill state (Fig. 4C). Confidence was lowest near the boundary, and highest at the extreme (centre, or edges). This result provides additional, albeit indirect, evidence for the interpretation that self-attribution bias operates also at a perceptual level.

In the discussion, first additional paragraph (p. 17, l. 567-573):

However, we also detected a strong effect of hit location on confidence reports (Fig. 4C). In short, confidence was lowest near the model-estimated subjective threshold between centre and edge, and highest at the extreme (centre, edges). This result provides an additional line of evidence therefore that self-attribution bias might operate also at a perceptual level. Had the threshold parameter not reflected a perceptual feature but simply a general appraisal of task difficulty, confidence would have shown a different pattern, i.e., decreasing with distance from the centre.

In the discussion, second additional paragraph (p. 18, l. 592-603):

Ultimately, while we interpret the subjective threshold parameter $\boldsymbol{\theta}$ as reflecting participants' internal estimate of their own motor precision, we acknowledge that it can also be viewed as encoding beliefs about task difficulty—i.e., the boundary between good and bad performance. Teasing apart perceived ability from inferred task demands is inherently challenging, as both likely contribute to the setting of $\boldsymbol{\theta}$. However, several findings support our interpretation that perceptual distortions of one's own performance play a significant role. First, participants who overestimated their motor accuracy in the score prediction task also exhibited larger objective thresholds during the main task (Supplementary Fig. X), underscoring the positive correlation between motor ability and perception of motor ability. Second, confidence ratings systematically tracked the subjective threshold (Fig. 4C): confidence was lowest near the threshold and highest near the extremes, consistent with an internal sensory-based representation rather than purely normative inference about task rules.

2. The authors frame the modelling as designed to disentangle two different underlying cognitive processes—a perceptual one and a feedback processing one. But I think it is not sufficiently clear how exactly this question translates into their modelling approach. I suggest pointing this out when describing the parameters and when reporting the model comparison results. As it is, model comparison is presented more as something performed for completeness than because it is essential for answering the question, which I think can be confusing.

This is a fair point. In this revision, we aimed to make a more apparent distinction between the two underlying cognitive processes at the model level by explicitly discussing model parameters. Additionally, and equally importantly, we also discussed the model comparison results more in-depth since they play a substantial role in our ability to disentangle these competing interpretations.

We made the following modifications to the main text:
(1) Added explicit mappings between model parameters and underlying cognitive processes
(2) In the results section, we discuss the model comparison results more in-depth
(3) Finally, in the discussion section, we better explain how the results obtained (from behaviour, model fitting, and model simulations) favour one over the other cognitive process

See below for excerpts from the main text relating to each point.

results, model introduction (p. 6, l. 201-220):

To test these two possibilities, we developed a computational model of our task in which feedback derives from the joint effect of the hit location (i.e., distance from the centre of the mole) and the hidden task state (Fig. 2B). The model simulates participants' beliefs about the hidden state using a leaky error-evidence accumulator updated on every trial by an error term computed from the mismatch between the expected hit location (centre, edge) and the feedback obtained (positive, negative). Within the model, the more positive the accumulated belief value is, the more likely participants will choose the random state.

After the agent strikes a mole at a distance dt from its centre, the model compares this distance to a subjective threshold θ. If dt < θ, it expects positive feedback; otherwise, it expects negative feedback. A binary error signal et is generated—0 if the actual feedback matches the expectation, and 1 if it doesn't. This error updates the belief zt, weighted by a sensitivity parameter α (which depends on the feedback type), and modulated by an integration factor γ to discount prior beliefs. Finally, the probability of a latent state is computed by applying a sigmoid function to the updated belief with a feedback-dependent constant term β. Differences in error sensitivity α or constant term β between positive and negative feedback would suggest a feedback/decision-dependent mechanism of self-attribution bias. Instead, perception of motor ability is captured by the parameter θ, modelling participants' subjective threshold, i.e.

the perceived boundary coding a switch in feedback from positive to negative in the 'skill' state. A threshold θ larger than the true threshold would indicate an inflated perception of motor ability.

-results, model comparison (p. 8, l. 270-281):

Our model comparison analysis further strengthened these results. We compared the full model with alternative, simpler versions. In particular, we were interested in the direct comparison with a single feedback-independent error sensitivity or constant term, true threshold, and no retention factor. The full model better accounted for participants' choice strategies (mean AIC: 463.9, see Supplementary Fig. 5). In addition, the model comparison further highlighted that the perceptual inflation (subjective threshold θ) played a larger role than overall positivity bias (error sensitivity α and constant term β) in determining behaviour, given the larger AIC difference from the full model when this parameter was fixed to the true threshold value ("true threshold", mean AIC: 525.5) than when either error sensitivity or constant term were set to symmetric across feedback type ("common error sensitivity", mean AIC: 483.3; "common constant term", mean AIC: 502.0, Supplementary Fig. 5).

discussion (p. 15, l. 479-484):

Simulations and model comparison analyses validated this result and rejected alternative interpretations. First, the model with a fixed threshold was significantly worse than the model with a subjective threshold at explaining participants' data ($P < 0.001$, average ΔAIC = 61.6). Second, the model with a fixed threshold was also significantly worse than alternative models with feedback-independent error sensitivity ($P < 0.05$, average ΔAIC = 23.5) or constant term ($P < 0.001$, average ΔAIC = 42.2).

3. The authors state that 'Thus the parameter fitting highlighted the overall tendency for positive scores to lead to skill choices', because alpha_pos was larger than alpha_neg and beta_pos was more negative than beta_neg. However, this doesn't seem to be quite the case, since the two parameters push the decision in different directions. While a more negative beta means pushing towards skill, a larger positive alpha (which is the case here) increases z and therefore pushes the decision towards random (again, assuming I understood correctly the model). And there seems to be a negative correlation between alpha and beta for each feedback type, according to Figure 2D. I think it would be useful to discuss this in more depth.

We apologise for the confusion. The statement quoted by the reviewer referred specifically to the model fitting results for the beta parameters. In those results, we found that beta_pos was more negative than beta_neg, thereby biasing the decision towards the skill state. Separately, it is correct that a larger alpha_pos increases the accumulated evidence z, pushing the decision towards the random state.

In both the task and the model, errors following positive feedback could only occur in the random state (and, for a small number of participants with a subjective threshold theta lower than the objective threshold, also in the skill state). As such, positive feedback errors were likely less ambiguous than negative feedback errors—which could arise in either state—resulting in higher sensitivity to positive errors, reflected in a larger alpha_pos parameter. In the model, a larger or smaller alpha modulates the accumulation of error, expanding or reducing the upper bound of the belief update. However, the lower bound remains fixed at beta, and the decision boundary is at zero.

This leads to a functional trade-off between alpha and beta for each feedback type: while alpha biases belief updates towards the random state, beta pushes them towards the skill state. Importantly, this is not a case of parameter redundancy but an interaction between distinct processes. If the two parameters reflected the same underlying mechanism (i.e. if there were information leakage), we would expect poor recoverability in model fitting and parameter recovery analyses—which was not the case.

We have now added these points in the discussion (p. 16-17, l. 527-545):

Our model formalises this dynamic using two parameters per feedback type: α, modulating error accumulation, and β, biasing beliefs toward the skill state. These parameters exert opposing forces: larger α increases the accumulated belief evidence in favor of the random state, while more negative β shifts belief towards skill attribution. While our parameter fits revealed that αpos > αneg (suggesting higher learning from surprising successes), this was accompanied by βpos < βneg, indicating a concurrent attribution bias toward skill in positive feedback (since this parameter shifts the baseline of the fluctuation regardless of the existence of error). These opposing forces likely balance each other in shaping choices, and indeed, we observed a negative correlation between α and β within each feedback type. This reflects a trade-off between error sensitivity and prior bias, not parameter redundancy. If both parameters encoded the same latent process (i.e., if there were information leakage), they would not be independently recoverable—a possibility ruled out by our model recovery analyses (Supplementary Fig. 8).

Additionally, we found a positive correlation between $\boldsymbol{\theta}$ (the subjective accuracy threshold) and αpos, suggesting that participants who overestimated their precision were more likely to interpret unexpected successes as random events. In this view, αpos might act as a compensatory mechanism n which higher αpos corrects for inflated self-beliefs. Overall, our findings expand on prior work by showing that attribution biases may emerge not only from motivational factors, but also from structural features of the task and distortions in self-perception.

I think this will also be relevant for the paragraph about the alpha parameter in the discussion, which I found a bit confusing. First of all, the authors state that Zamfir & Dayan's finding about participants updating their beliefs more after outcomes attributed internally than after those attributed externally aligns with the higher alpha for positive scores, which they say 'indicate that people tend to credit themselves for successes more readily than attribute failures to their actions'. If I understand the model correctly, this is not quite what the alpha parameter indicates: it shows how much participants weigh evidence in favour of 'random', implying the opposite of what they claim in this paragraph.
It might also be relevant to mention here that Zamfir & Dayan found a larger learning rate for negative than positive feedback, which differs from the authors' results.

Thank you for bringing up this inconsistency. We have rewritten the paragraph accordingly and included the results from Zamfir & Dayan referred to by the reviewer (i.e., that participants displayed a larger learning rate for negative than positive feedback).

(p. 16, l. 506-526):

A recent study revealed dynamic, reciprocal links between outcome attributions and self-beliefs, consistent with the attribution-self-representation cycle theory (Zamfir & Dayan, 2022). Participants were more likely to update their beliefs about their abilities when they attributed outcomes to themselves rather than external factors. With increasing skill estimates, participants took more credit for wins and less blame for losses. The authors also found a higher learning rate for negative than positive feedback. Similarly, Mancinelli et al. (2024) showed that internality is specifically linked to enhanced learning from failure. A different set of studies also reported greater error sensitivity following negative outcomes, driven by a self-attribution bias, as participants needed good results to earn rewards and thus learned more from failures (Dorfman et al., 2019, 2021).

In contrast, our study found greater error sensitivity (α) for positive feedback. This difference likely stems from our task structure: participants had to infer the task state (random or skill) from probabilistic, implicit feedback. Negative feedback was ambiguous—arising from both motor errors in the skill condition and

accurate actions in the random condition—whereas positive feedback in the random state clearly signalled a mismatch between belief and outcome. As outcomes were only partially informative of hit location, and further obscured by motor noise, participants might have weighted more diagnostic (positive) feedback more strongly. Thus, our study highlights how task structure and outcomes interact with self-attribution bias to shape error assignment and learning, which may also underlie apparently opposing results (Kim et al., 2019; Lee et al., 2024).

Mid-level:

1. in the main text, the authors report that participants were above chance in detecting the condition (Skill vs random). However, the interpretation of this information changes when one finds out that they excluded people who were below chance in detecting the condition (as per the methods). I think making this claim is somewhat misleading; at the very least, I believe the authors should report the exclusion criteria and the statement about the inference accuracy in the main text.

We agree and now report results and statistical testing for performance above chance using all participants' data (N=66). As such, we replaced Fig. 1c to show all participants' data (see new panel, which we added below, with dots indicating included participants, and crosses indicating excluded participants), and report the accuracy before exclusions in the main text. Note that results remain unchanged.

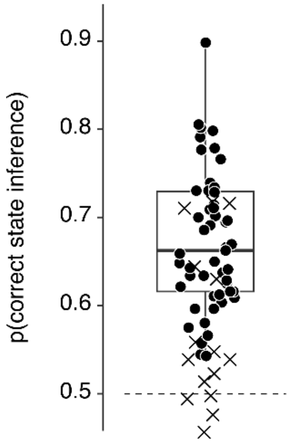

We have added the following text to the manuscript (p.4, l. 112-116):

[...] participants' (N = 66) inference accuracy was above the theoretical chance at the group level (mean accuracy 0.65 ± 0.10, Wilcoxon signed rank test against chance level 0.5: Z = 6.63, $P < 0.001$). Participants with at-chance performance or fixed confidence ratings were excluded from further analyses (see Methods for full exclusion criteria). Thus, N = 51 participants were included in all subsequent analyses.

2. The fact that inference accuracy was higher following positive scores is surprising, assuming the task was designed such that participants would get positive and negative feedback in roughly half the trials in skill, and with matching frequency in random. The authors address this by performing an ANOVA analysis, but I think it helps to have finer-grained information about what feedback is given in what proportion of trials, in both conditions, in the main text. This information is indeed in the supplementary

materials. Still, the way it is mentioned, very in passing, in the main text, seems a bit misleading to me: towards the end of the first paragraph of the introduction the authors say 'The task was calibrated such that participants would obtain positive scores with similar probabilities in both task states', which seems to imply this calibration was successful. However, supplementary Fig. 2 shows this was not the case. There was, in fact, a significant difference in the mean at the population level, and the distribution across subjects in the two conditions was qualitatively very different. Given the large amount of variability in the proportion of positive feedback in the skill condition, I think it would be helpful to see if there is any relationship between the proportion of positive feedback in the skill condition and the proportion of correct inference across subjects. Because the pattern or feedback x state interaction for accuracy -higher accuracy after negative feedback in the random state and higher accuracy after positive feedback in the skill state- is compatible with a pattern where participants would roughly choose 'skill' after positive feedback and 'random' after negative feedback.

We thank the reviewer for their thoughtful comment aimed at further disentangling the contribution of feedback valence to inference accuracy. The reviewer is correct in noting that the probability of receiving positive feedback in the skill state varied across participants and was, on average, higher than in the random state. We would also like to emphasise that the feedback-response mapping pattern—'positive feedback → skill choice' and 'negative feedback → random choice'—aligns with our own interpretation.

In line with the reviewer's suggestion, we computed the correlation between the proportion of positive feedback received in the skill state and the proportion of correct inferences. This analysis revealed a significant positive relationship (regression slope = 0.24, t_{49} = 3.35, P = 0.002; see figure below, now added to the supplementary materials). This finding supports the notion that a higher frequency of positive feedback in the skill state is associated with greater overall inference accuracy, consistent with a general mapping of positive feedback to skill-based choices.

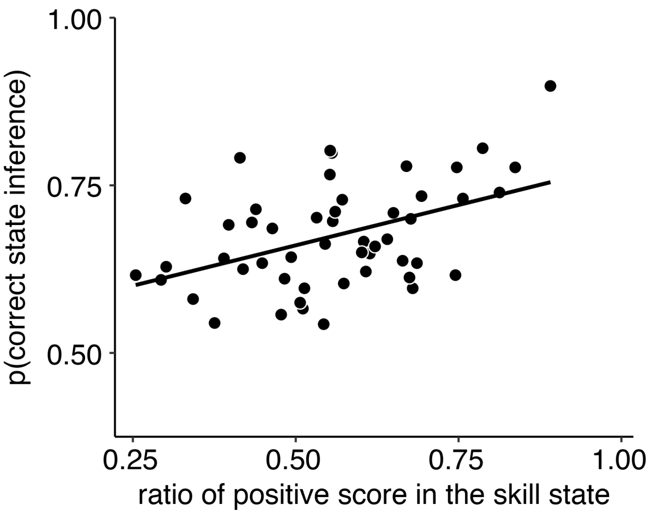

3. I think in the current version of the manuscript, there can be some confusion for the reader as to what self-attribution bias is: it seems to be defined as equivalent to self-serving bias, i.e. the tendency to attribute mistakes to outside causes and successes to oneself. However, in the next paragraph, the authors say it has been linked with depression, which is confusing: it would be better, I think, to make clear that depression has been linked with a reduced self-serving bias. Then the authors quote Zamfir & Dayan as proposing that self-attribution might give rise to learned helplessness; however, this is not what

the paper suggests. On the contrary, Zamfir & Dayan found a self-serving bias which might have a protective effect, counterbalancing higher learning from negative feedback.

We thank the reviewer for pointing out this inconsistency in the introduction. By self-attribution bias, we indeed meant the tendency to attribute mistakes to outside causes and successes to oneself, as in self-serving bias. We have revised the relevant sentences accordingly (see below, from p. 2, l. 62-67):

In the context of mental health, depression has been linked with reduced self-attribution bias and the development of learned helplessness (Abramson et al., 1989; Seligman, 1972; Seligman & Maier, 1967). A recent study proposed self-attribution bias as a computational mechanism that might protect against learned helplessness, counterbalancing higher learning rates from negative feedback (Zamfir & Dayan, 2022).

4. The instructions and information given to participants in this context are likely to matter a lot: as the authors say, 'whack the mole' is generally perceived to be a skill task. Therefore, it matters how participants are informed about the random condition. The methods are not clear and detailed enough to find out exactly how participants were instructed. Also, I think describing feedback as 'positive or negative score' might suggest numerical values, rather than 'Good' or 'Bad'. I think it would be better to use a different term in order to avoid confusion.

We have added the complete set of instructions given to participants in each task phase in supplementary materials (see pp. 19-20). Second, following the reviewer's useful suggestion, we have changed the term ‘score’ throughout the manuscript to ‘feedback’, which we think is better suited to the context of our task.

5. I think the description of the model and assumptions it relies on (e.g, participants have access to the true distance between hit location and target centre, if I understood correctly) needs to be clearer in the main text. As it is, I don't think one can interpret the results reported in the main text about the model parameters without reading the methods for additional details. I have two suggestions here: 1. to walk the reader through what the model does on every trial (ex. the participant keeps an estimate of x, transforms that into y, which, together with parameter k, controls action; once they receive feedback they compute z, and update x with z weighted by parameter b etc) And or 2. explicitly say what the different parameters do: e.g. a high value of parameter A means that subjects will do x more than if the value is low etc. This does not need to be very long, and would make the text more readable.

We have followed the reviewer’s helpful advice and rewritten the paragraph describing the model and the underlying assumptions (p. 6, l. 209-220):

After the agent strikes a mole at a distance dt from its centre, the model compares this distance to a subjective threshold θ. If dt < θ, it expects positive feedback; otherwise, it expects negative feedback. A binary error signal et is generated—0 if the actual feedback matches the expectation, and 1 if it doesn’t. This error updates the belief zt, weighted by a sensitivity parameter α (which depends on the feedback type), and modulated by an integration factor γ to discount prior beliefs. Finally, the probability of a latent state is computed by applying a sigmoid function to the updated belief with a feedback-dependent constant term β. Differences in error sensitivity α or constant term β between positive and negative feedback would suggest a feedback/decision-dependent mechanism of self-attribution bias. Instead, perception of motor ability is captured by the parameter θ, modelling participants' subjective threshold, i.e. the perceived boundary coding a switch in feedback from positive to negative in the ‘skill’ state. A threshold θ larger than the true threshold would indicate an inflated perception of motor ability.

Further minor notes about the model description:

-it would be helpful to clearly state what the evidence being accumulated in the model is about: i.e. that it is treated in the model as evidence about the task being in the 'random' state.

Thanks for the suggestions--we have now added this to the main text, as follows (p. 6, l. 204-208):

The model simulates participants' beliefs about the hidden state using a leaky error-evidence accumulator updated on every trial by an error term computed from the mismatch between the expected hit location (centre, edge) and the feedback obtained (positive, negative). Within the model, the more positive the accumulated belief value is, the more likely participants will choose the random state.

-in the model graph in Figure 2B, I find the labelling of variables as 'model' vs 'observed' somewhat confusing: s_t is grey but is not in the model variables rectangle, s^hat_t is in the model variables rectangle, but not in the observed variables, even though it is observed by the experimenter.

We have updated the figure to increase its clarity. We have renamed 'model' variables to 'internal' variables, reassigned choice s^hat_t to the 'observed' variables, and changed the colours to ensure uniformity across levels.

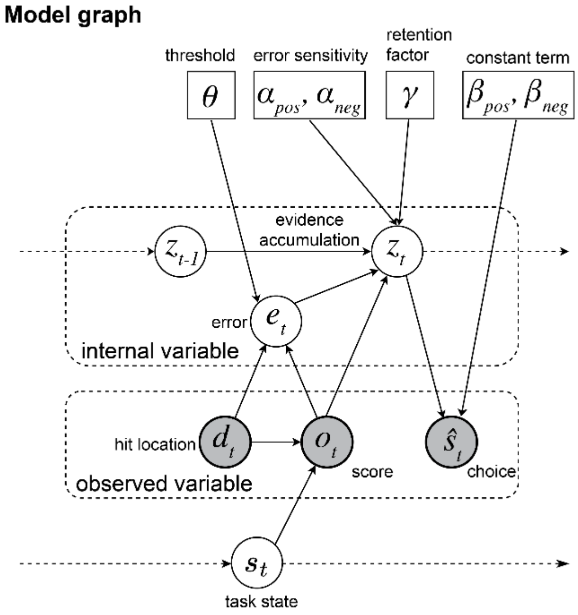

Low level:

1. Second phrase in the third paragraph of the introduction, starting with 'Similarly, in educational settings': the authors seem to want to switch from the students' perspective to teachers' perspective, but something is missing.

Thanks for catching this. We have rewritten the sentence as follows (p. 2, l. 56-60):

Similarly, in educational settings, students often credit high grades to their own intelligence and effort while teachers attribute these successes to their own effective teaching methods, whereas students blame poor grades on unfair tests while teachers blame inadequate student preparation (Covington, 1992; McAllister, 1996).

2. Supplementary figure 3B: Does this only refer to the 'skill' condition? It should, I think, but it is not clearly stated.

Sorry for the confusion. Supplementary Figure 3B refers to the whole dataset. This is now written explicitly in the figure legend.

3. Last paragraph before section "Behavioural signatures of self-attribution bias and computational modelling": 'The magnitude of the difference was larger after a positive score compared to a negative score, whichever rule they chose." This is not clear to me: is this the difference between the distance to the centre in trial t+1 vs trial t? Because it doesn't seem to match the pattern in Sup Fig 4C.

There was a mistake, which has now been corrected, as follows (p. 6, l., 185-186):

Participants hit closer after negative feedback than after positive feedback, whichever rule they chose (random: Z = -5.32, P_{FDR} < 0.001; skill: Z = -6.21, P_{FDR} < 0.001).

4. Third paragraph of the 'Behavioural signatures of self-attribution bias and computational modelling' section: 'We developed a computational model of our task in which scores derive from the joint effect of the hit location and the hidden task state'. I think it should be made clearer that this is a model of subjects' beliefs about how scores are derived.

Thanks for the suggestion. We have rewritten the sentence as follows (p. 6, l. 204-207):

The model simulates participants' beliefs about the hidden state using a leaky error-evidence accumulator updated on every trial by an error term computed from the mismatch between the expected hit location (centre, edge) and the feedback obtained (positive, negative).

Reviewer #2:

I commend the authors for their innovative approach to investigating self-attribution bias. Through a carefully designed experiment, they tried to not only document the existence of this bias but also propose and validate an underlying mechanism driving it. I appreciate the authors' effort to deviate from conventional experimental designs and their thorough modelling analyses.

We thank the reviewer for their positive endorsement of our study.

However, I have several concerns regarding the task design, the potential influence of confounding factors, and the validity and robustness of their modelling analyses.

Data Filtering:

I am concerned about the authors' decision to pool data from all confidence levels without differentiating between them. Initially, I believed that the confidence feature was intended as a filter, allowing responses with low confidence (levels 1 and 2) to be excluded from analysis. While the authors separately discuss the confidence data in their results, pooling choices from low-confidence trials (basically guesswork) with high-confidence choices is problematic. The analysis would benefit from excluding low-confidence data and reconsidering how these responses are treated in the regressions, tests, and modelling analyses.

Thank you for this suggestion. We agree that confidence level is an important feature of the data; however, precisely because our goal is to characterise how participants' subjective uncertainty varies across the entire confidence spectrum, excluding 'low-confidence' trials would undermine our central objectives and distort our estimates.

Classical signal detection–theoretic measures of metacognition (e.g., meta-d′ and M-ratio) rely on the full distribution of confidence ratings to disentangle sensitivity from bias. If one removes all trials rated 1–2, one effectively truncates the lower tail of the confidence distribution, artificially reducing variance and statistical power and biasing metacognitive efficiency estimates upwards (Maniscalco & Lau, 2012).

Virtually every influential study of decision confidence—from the demonstration that confidence behaves like a Bayesian posterior probability (Meyniel, Sigman & Mainen, 2015) to work on post-decision biases (Luu & Stocker, 2016)—includes the full range of confidence ratings. Truncating the data by filtering out "guess" trials has been shown to inflate estimates of metacognitive precision and can even reverse observed effects, since low-confidence errors often carry diagnostic information about uncertainty (Meyniel et al., 2015; Luu & Stocker, 2016).

Low-confidence responses index genuine subjective uncertainty and carry crucial information about participants' calibration and resolution (Koriat & Goldsmith, 1996). Indeed, changes in the distribution of low versus high confidence ratings have been linked to dissociable shifts in both calibration (overall bias) and resolution (discrimination between correct and incorrect trials) (Koriat & Goldsmith, 1996).

In any SDT model, confidence (i.e., the inverse variance of the predicted probability of a correct response) is estimated across the entire scale. Removing levels 1-2 would collapse the very variance that informs our estimates of metacognitive ability and bias, leading to misleading parameter values.

For these reasons, pooling across all confidence levels is both appropriate and necessary to achieve our study's aims. We have now clarified these points in the text.

Task Design:

The primary observed effect of self-attribution bias is that participants are more likely to attribute their performance to "skill" when receiving a positive score. However, it remains unclear whether self-

attribution bias is the only or primary driver of this effect. A key assumption underlying the authors' conclusions is that participants perceive a 50% chance of success in “skill” sessions, which is the same as in “random” sessions. However, I am sceptical that this condition is met, and the observed effect might be driven by inference bias, prior beliefs, and psychological accounts:

• Inference Bias: Participants have no idea of the threshold and have to infer it. Given the way they design the task, I would be truly surprised if participants accurately inferred the threshold. Naturally, the observed effect could be because they inferred the threshold to be larger than the true value, so the probability of winning is >50% in “skill” sessions, but this may not necessarily be due to self-attribution but simply some inference biases. I am wondering if some well-documented inference biases can explain the overestimation of the threshold. If so, what they observed is an existing inference bias which manifests as “overconfidence” in this task.

We thank the reviewer for raising this important point. To clarify, “inference bias” is often used broadly to denote any systematic deviation from statistically optimal belief-updating, whether confirmation bias, optimism bias, conservatism (under-updating), the base-rate fallacy, or indeed self-attribution bias. In that sense, our self-attribution effect is itself a case of inference bias: participants overestimate the performance threshold in the “skill” sessions because they attribute past successes to their own ability. This distorted inference leads them to believe the threshold must be higher than it really is, yielding a win probability in excess of 50%.

Prior Beliefs from Training: The task is cognitively demanding: they must actively infer exactly where they just hit and where the threshold is in each trial. They will likely use more simplified approaches, such as forming a belief about their probability of winning in the skill state and using this belief to make inferences. In this case, the training phase, where participants are exposed to customised thresholds, could contribute to forming optimistic prior beliefs.

o If many participants achieve greater than 50% success during training, they may carry this belief into the main task, resulting in the attribution of “skill” following positive feedback.

This is an intriguing suggestion. Note that during the practice, participants received feedback on their inference choices. We first confirmed that most participants achieved greater than 50% success during training (50 out of 51 included participants, see left-side figure below). Following this comment, we also found no relationship between the rate of success in training and the size of the self-attribution bias (measured as reported in the main manuscript, as the ratio between subjective and true threshold, see right-sided figure below), suggesting that this alternative explanation is not supported by the data.

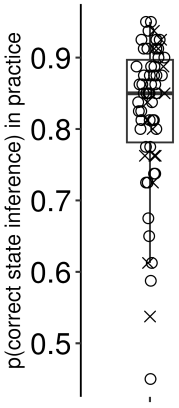

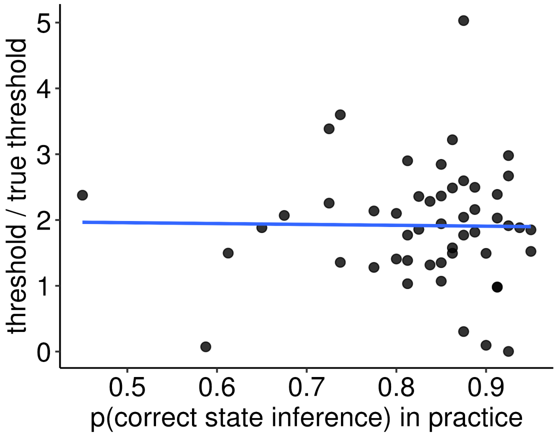

o The authors attempt to justify this possibility with Supplementary Figure 2, but I find the graph unconvincing:

▪ The upward skew and large volatility observed in the data suggest that an optimistic prior belief is a reasonable assumption for most participants.

The plot in Fig. S2 shows a wide range of task performance, with an average rate slightly above 50%. It is unclear why this population-level variability should translate into an optimistic prior belief.

▪ Such post-game stats are not relevant to participant belief. We don't know how and if participants updated their beliefs or what beliefs they had.

We thank the reviewer for this comment. It is unclear what is meant by "post-game stats," since all statistical analyses are conducted after data collection. Moreover, our analysis was never intended to "access" participants' inner beliefs (an endeavour we consider infeasible in any cognitive-task analysis). Rather, it was performed solely to demonstrate the calibration procedure and estimate performance variance—an approach routinely employed in tasks of this nature. While the original scope did not include testing whether the calibration procedure itself induced the self-attribution bias, we have, as per the reviewer's suggestion, now examined this possibility and found no relationship between calibration-level performance and the magnitude of the effect we report.

▪ Supplementary Figure 2 legends: "Wilcoxon signed-rank test, $Z = 2.58$, $p = 0.01$". The authors should have conducted a two-sided test here. If the conclusion is that the scores are similar across states (they fail to reject the null hypothesis), shouldn't the p-value be close to 1 instead of 0?

We would like to clarify that the original statistical test reported in supplementary figure 2 was two-sided. Also (as mentioned before), our goal was not to reject the null hypothesis as the reviewer seems to imply.

• Ease of Performance Evaluation: The graphical design of the task may unintentionally bias participants. It appears easier for participants to evaluate very good motor performance than very poor performance. Note that this is different from what the authors discussed on page 5: not that positive scores easier to evaluate, but good motor performance is easier to recognise. This difference in ease of evaluation could

explain the observed effect. To examine this, the authors could consider filtering the data to focus on only trials with high confidence (level>=3) and unambiguous motor performance, and examine whether the observed effect is because there are more trials where participants performed unambiguously well. If yes, the observed effect is due to the task design and psychological accounts instead of self-attribution bias. Otherwise, if the "low performance" group dominates and we still observe the effects among the filtered data, it would be more compelling evidence of self-attribution bias.

We thank the reviewer for their suggestion. However, there appears to have been a misunderstanding regarding the task. In our study, confidence pertains to inference rather than motor performance, and therefore, the suggested analysis is not applicable. Additionally, we do not see a clear reason why participants would find it easier to evaluate very good versus poor performance or how such an evaluation could systematically bias performance in a way that would account for the effect we report.

• Incentives and Asymmetric Effort: The bonus feature, which rewards participants for correctly estimating the task state and achieving a positive score, could also introduce bias. Since no reward is given for correctly estimating the state with a negative score, participants may exert less effort during these trials, skewing the results in favour of the "skill" state.

This paper explores how individuals attribute errors to poor performance or chance. To investigate this, task feedback must retain a degree of ambiguity. If correct inferences were rewarded regardless of actual scores, the task would become trivial, as participants could immediately identify the underlying state. Moreover, because hidden state inferences were continuous and trials were not independent, it is unlikely that participants would reduce their effort following negative feedback. Doing so would compromise their chances of obtaining rewards in subsequent trials. In fact, we observed that participants consistently adjusted their responses based on feedback: after negative feedback, they tended to aim closer to the centre, and after positive feedback, they aimed further away (see Supplementary Fig. 4C, shown below). This pattern reflects a standard motor adaptation effect, in which participants make subtle, trial-by-trial adjustments in response to outcome information.

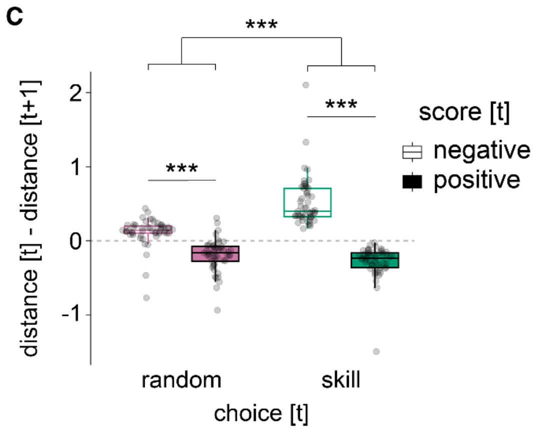

• Performance Volatility and Sample Size Justification: The volatility of participants' performance raises concerns about the sample size and the power of the analyses. Without justification of the sample size or a power analysis, the robustness of the results is uncertain. Furthermore, I presume that no preregistration was conducted.

We are not entirely sure what the reviewer means by "volatility" in this context—perhaps they are referring to variability? The trial-to-trial variation observed in participants' scores is well within the range reported in

established motor-control studies (e.g., Wolpert et al., 1995; Shadmehr & Holcomb, 1997). In such paradigms, endpoint errors in reaching tasks typically show a standard deviation of 5–10% of movement amplitude, reflecting the inherent noise in sensory feedback and motor commands. If our task had produced virtually no variability, it would have indicated that the manipulation was either too simple or too difficult, undermining its sensitivity to the attribution processes under investigation.

Normal behavioural variability does not compromise statistical power; on the contrary, it provides the dispersion necessary to detect shifts in central tendency, such as median thresholds. To mitigate any risk of under-powering, we conducted an a priori power analysis using G*Power 3.1. This analysis, based on a target effect size of Cohen's d = 0.5 (a medium effect commonly reported in motor-control research; see Krakauer et al., 2000), $\alpha = 0.05$, and $1–\beta \geq 0.80$, indicated a required sample size of N = 34 for within-subjects' comparisons. We ultimately recruited N = 51, exceeding this threshold by over 40%. All key comparisons are reported with effect sizes, exact p-values, and corrections for multiple comparisons. In every case, our results fall well below standard significance thresholds used in similar behavioural studies (e.g. Cohen, 1988; Lakens, 2021). We therefore believe that the normal performance variability we observe enhances our design's sensitivity, rather than detracting from it.

Modelling Setup:

• I was surprised to see that the prediction error in the model was treated as binary. The assumption that participants clearly know whether they hit inside or outside the threshold is unrealistic, given that the participants may not have actively inferred the threshold and their hit location. Moreover, the accuracy of the hit (i.e., how close it is to the threshold; dt-theta in the models) should matter. For example, a hit near the threshold would feel ambiguous to the participant, and the prediction error would likely be smaller regardless of the results. The model's treatment of prediction error as binary may not accurately reflect the nuances of cognition.

We chose to use binary prediction error (PE) precisely for the reason the reviewer highlights: participants are not aware of the exact location where they hit the mole. We opted to use binary prediction error (PE) for the very reason the reviewer points out: participants do not have access to the precise location where they hit the mole. In our model, the error signal is derived from the theta parameter, which represents the subjective threshold where participants believe the central, rewarding area lies in the skill state. This approach does not require explicit knowledge of the threshold's position. Instead, it assumes participants maintain an internal estimate of where this boundary might be, and we use the fitted threshold parameter to compute the binary error. By modelling the error as binary, we incorporate this inherent uncertainty and avoid relying on exact hit positions. The alternative would require participants not only to infer their hit location with precision but also to maintain a monotonic representation of their distance from the threshold—an assumption we view as highly implausible.

This is now explained in clearer terms at the beginning of the results where we introduce the model (p. 6-7, l. 220-226):

Note that while the model assumes that participants have limited access to the precise hit location, this assumption is restricted to a binary distinction—whether the hit landed within the centre area or outside it. The model does not otherwise rely on precise spatial information (i.e., the exact distance from the centre), which participants could not access. However, it does incorporate the idea that participants have an internal representation of the centre area—delimited by the hidden threshold—and are therefore able to form a general sense of whether a hit was relatively close to or far from the centre.

Incorporating Confidence into the Model: While the authors tried to justify this, the model does not appear to incorporate confidence levels into the decision-making process or the model fitting. Ideally, confidence could be generated jointly with the choice data, reflecting the participant's certainty about their performance. This might be achieved by incorporating the accuracy of the hit; see above. If such modelling is intractable, the least thing to do is to incorporate the confidence data into model fitting, e.g., if the confidence in each trial is high, more weight should be given to the LL in that trial.

We would like to clarify a few points. First, we did not seek to justify the exclusion of confidence from the model—this was a deliberate modelling choice. Our intention was simply to note that, although confidence was not explicitly modelled, the framework still successfully captured several key features of participants' confidence judgements. We believe this strengthens the case for the explanatory power of our approach. Second, as noted in a previous response, there appears to be a misunderstanding regarding the nature of confidence in this task. Here, confidence does not reflect participants' certainty about their motor performance, but rather their confidence in the underlying state. Finally, while we agree that it is possible to construct alternative modelling architectures that incorporate confidence (such as Kalman-filter-based models), this falls outside the scope of the present study. We do not believe such modifications would alter the core conclusions we draw from our results.

Modelling Analyses:

• Parameter Recovery: Many of the analyses rely on fitted parameters, which are known to be prone to uncertainty. While the authors provide some parameter recovery (Supplementary Figure 8), this does not fully validate their methodology. The correlation between parameters alone cannot justify the parameter recovery. A more rigorous validation would involve conducting simulations to assess the likelihood of false positives and false negatives. For instance, one could simulate choices without bias (e.g., assuming no difference in alpha between positive and negative feedback) and then fit the model to see if the results lead to false positives (detection of a higher alpha for positive feedback). Similarly, simulations with varying levels of bias could assess the model's ability to detect biases without incurring significant false negatives. More professional robustness checks would involve varying the strength of the simulated biases and assuming that only a certain portion of agents is subject to the bias.

We fully agree with the reviewer that robust validation is essential for model fitting. We apologise for not making it sufficiently clear that we have, in fact, conducted multiple layers of validation, including simulations, parameter recovery, and now also model recovery. Simulations were carried out using both the full model and alternative variants to explore the range of behaviours each could generate and to confirm that participants' behaviour was best captured by the full model. Furthermore, as shown in Supplementary Figs. 6 and 11, we reported the correlations between participants' observed behaviour and the model-simulated values, which further support the validity of our modelling approach and conclusions. We have included results of these supplementary analyses below for the reviewer's convenience.

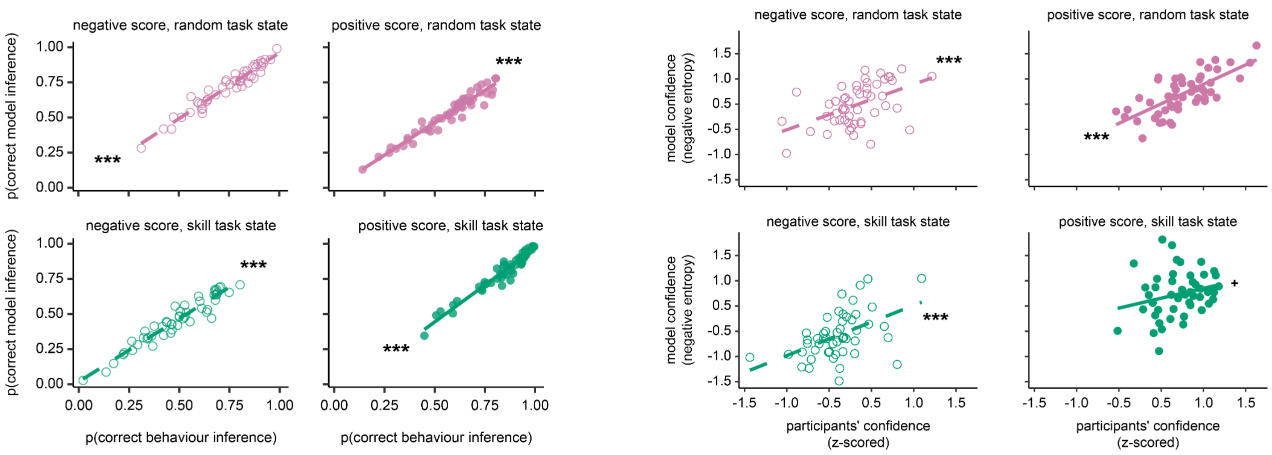
negative score, random task state
positive score, random task state
negative score, skill task state
positive score, skill task state
p(correct model inference)
p(correct behaviour inference)
model confidence
(negative entropy)
participants' confidence
(z-scored)
***
+

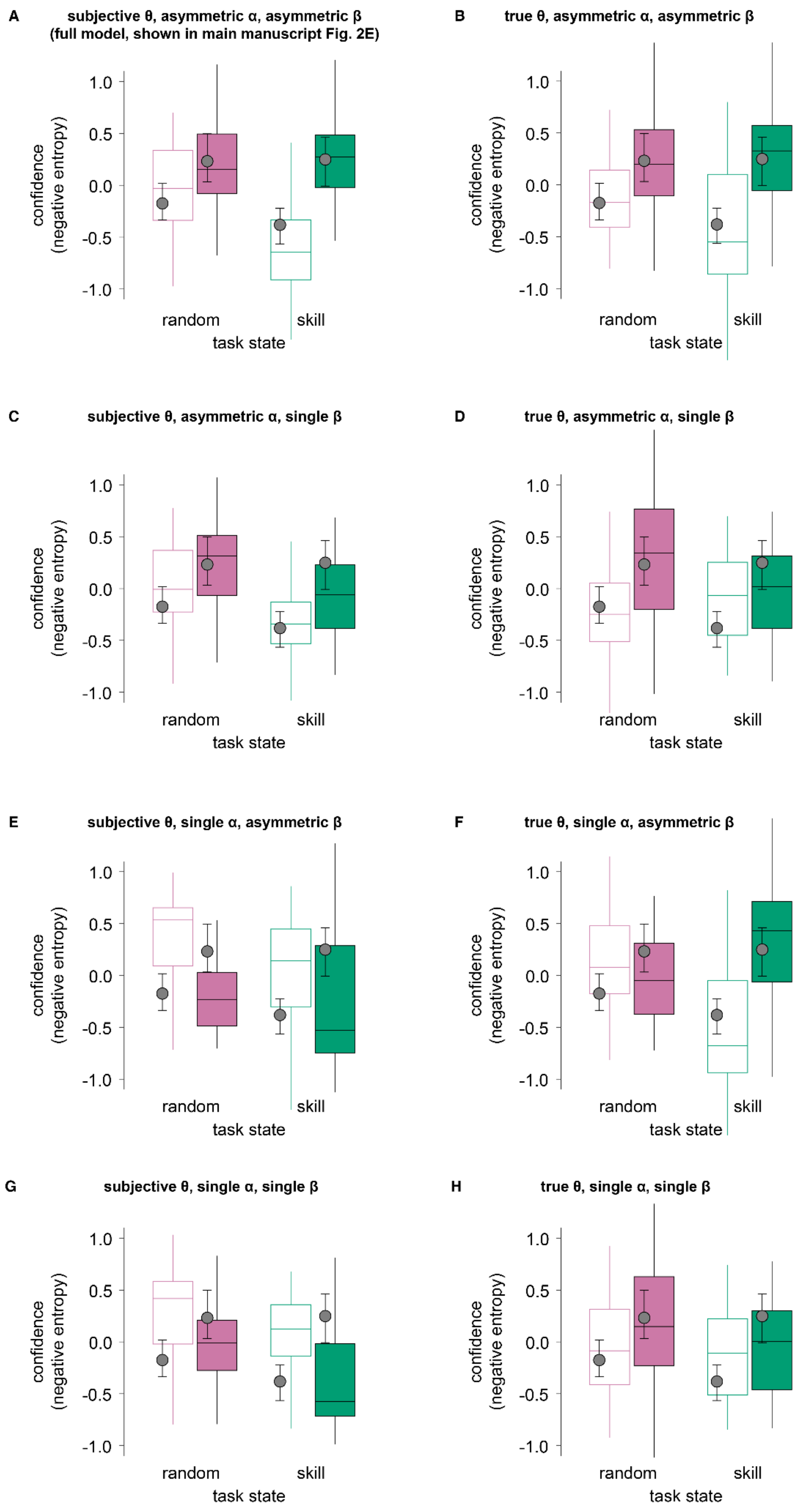
A
subjective θ, asymmetric α, asymmetric β
(full model, shown in main manuscript Fig. 2E)
B
true θ, asymmetric α, asymmetric β
C
subjective θ, asymmetric α, single β
D
true θ, asymmetric α, single β
E
subjective θ, single α, asymmetric β
F
true θ, single α, asymmetric β
G
subjective θ, single α, single β
H
true θ, single α, single β
confidence
(negative entropy)
1.0
0.5
0.0
-0.5
-1.0
random
skill
task state

• Model Comparison: Instead of relying solely on AIC and BIC criteria for model comparison, I suggest that the authors use cross-validation. This method would involve fitting the model to the first half of each participant's choices and then testing the model's ability to predict the second half. The performance of the models could then be compared based on out-of-sample likelihood. If the authors really want to keep the current comparison criteria, I recommend conducting a more thorough recovery analysis for all model variants: simulating the choices from one model variant and run model comparison, the true variant should outperform all alternatives.

We have conducted the model recovery analysis as suggested by the reviewer, simulating choices from each model variant and performing model comparisons. The results, now included in the supplementary materials as Supplementary Fig. 9, clearly show that the true generating model consistently outperforms the alternatives. It is worth noting that the full model always fits at least as well as the true variant. This outcome is expected, given that all compared models are nested within the full model—that is, each simpler model results from fixing one or more parameters of the full model. We find this pattern of results reassuring, as it supports the robustness of our modelling approach.

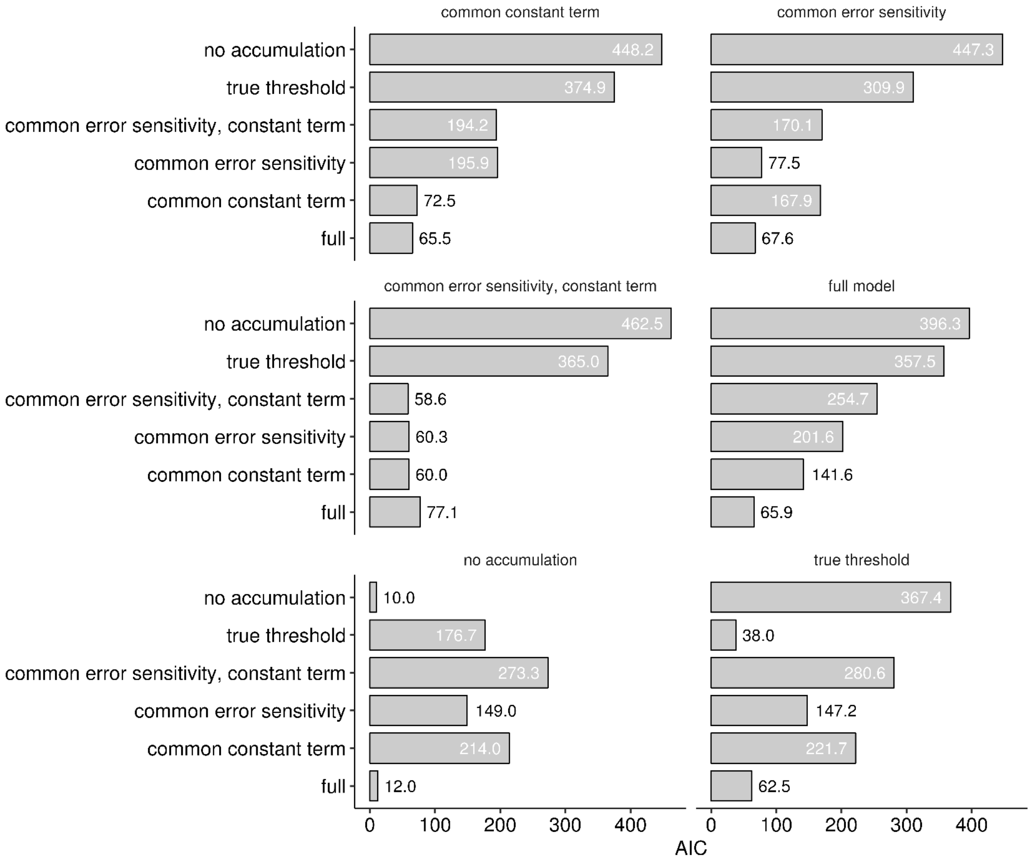

We have also added the following text in results (p. 9, l. 299-304):

Refitting simulated data with the entire set of models (full, simpler alternatives) highlighted the robustness of the model. The best fit model was the same as the underlying ground-truth, generative model (e.g., we recovered the full model when the full model had been used to generate simulated data, Supplementary Fig. 9). Together, these simulations, parameter and model recovery analysis demonstrate the robustness of the model, but also its ability to capture multiple contributing factors in participants choice behaviour and self-attribution bias.

Minor Comments:

• On page 5, to test the effect of the score, it is interesting to see whether the results are robust using the 100-point score instead of binary results. Participants in the game not only see the binary results but may also have a rough feeling about the accuracy of the hit, c.f., my previous comments.

Participants received binary scores in the main task (they only received graded scores for hits in the first pre-task training session). Indeed, we agree with the reviewer that participants not only saw the binary results but also had a rough feeling about the accuracy of the hit. This is well captured by participants' confidence rating, as predicted (see figure below). We have now added this new analysis to the manuscript (see new Fig. 4C).

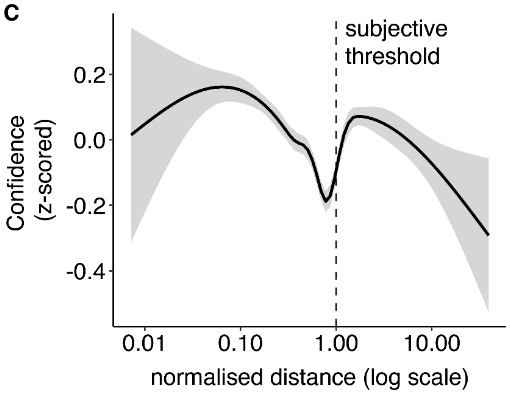

In the results, we added a paragraph reporting on this new finding (p. 11, l. 364-368):

Moreover, we found that confidence accurately reflected task uncertainty with respect to the subjective threshold parameter signalling a shift between positive and negative feedback in the skill state (Fig. 4C). Confidence was lowest near the boundary, and highest at the extreme (centre, or edges). This result provides additional, albeit indirect, evidence for the interpretation that self-attribution bias operates also at a perceptual level.

• On page 6: "Interestingly, we found that where participants hit the moles in relation to the centre differed depending on the objective task state (see Supplementary Fig. 4A-B, hits were closer to the centre in the skill state than the random state)." I thought this is not surprising. If their effort did not matter in the random state, of course the motor performance is worse. Not sure if I missed other interesting insights here.

Participants' effort was also relevant in the random state: correctly identifying positive feedback as originating from the random state resulted in a future reward. Indeed, participants adapted their hit locations similarly in random and skill conditions (see Supplementary Fig. 4C, included below). Following negative feedback, subsequent hits tended to be more precise, whereas positive feedback led to hits further from the centre. Crucially, this adaptation effect was observed in both conditions. We hope this clarifies the issue.

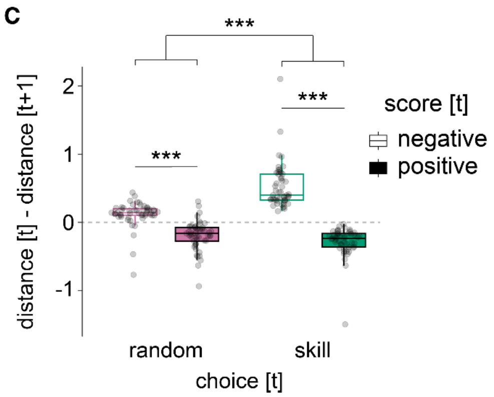

• On page 6: “Together, these results suggest that participants dynamically adjusted their hit location. However, there was no evidence that they did so strategically, in which case we should have seen an effect only in the ‘skill’ state and not in both, as reported here.” I don’t understand the logic here which seems to assume that participants can perfectly infer the underlying state. Participants didn’t know the true state and there could be significant lag effects in their inferences, so of course they would still implement their strategy in the random state.

We thank the reviewer for pointing this out and wish to rectify that the reported effect relates to *participants’ choices* and not hidden ask states (see figure in response to the comment above). As the reviewer rightfully notes, the latter would carry little meaning.

The related sentence in the main text has been updated accordingly (p. 5-6, l. 176-190):

[...] obtained feedback (negative, positive) and task state inference (skill, random) impacted where participants hit the mole on the following trial (Supplementary Fig. 4C, main effect of feedback $F(1, 50) = 159.68$, $P < 0.001$; main effect of inference choice $F(1, 50) = 24.96$, $P < 0.001$; interaction between inference choice and feedback: $F(1, 50) = 71.85$, $P < 0.001$). Participants hit closer after negative feedback than after positive feedback, whichever rule they chose (random: $Z = -5.32$, PFDR < 0.001; skill: $Z = -6.21$, PFDR < 0.001). Together, these results suggest that participants dynamically adjusted where they hit. However, there was no evidence that they did so strategically, in which case we should have seen an effect only in the ‘skill’ state and not in both, as reported here.

• The exclusion criteria should also account for participants with consistently low confidence (e.g., those with confidence level <3 for a large proportion of trials), as their data quality in in doubt.

Participants were excluded if they reported the same confidence level on more than 80% of trials. Introducing any additional exclusion criteria based on confidence would not be justified and would depart from standard practice in the field, potentially introducing bias into the dataset (see our response to point 1 raised by the reviewer on page 10 of this rebuttal letter).

• Page 27: “Finally, we calculated Spearman's rank correlation coefficient for each parameter and generated the corresponding confusion matrix (Supplementary Figure 7).” A typo, should be Figure 8.

We thank the reviewer for catching this error; it has now been fixed as suggested.

Reviewer #3: The paper touches on the question of attribution and its impact on further decision-making. It uses a new task with skill and random, hidden states, which differ in the way that feedback is given to subjects. The results are well developed and laid out. The approach is rigorous. I very much enjoyed reading this paper and do hope it will be published in this outlet.

This being said, there are some points that should be addressed to get this study in its best shape to be shared with the rest of the community. I'm going to lay these out in somewhat of an order.

We thank the reviewer for the positive assessment and for providing valuable suggestions to improve the paper's clarity.

1.a It seems that previous, very relevant work in the area is only very briefly touched upon and related to -- this goes for Mancinelli et al (2021); and Zamfir and Dayan (2024). The authors introduce their new task by "To resolve this question, we incorporated several new key features into our experimental approach (Mancinelli et al., 2021; Zamfir & Dayan, 2022). First, we diverged from previous studies by employing a sensorimotor task rather than the more commonly used multi-armed bandit tasks, in which factors beyond mere choice play no role."
The citation of these works is awkward here. Further nuance in this discussion is warranted -- esp. in relation to the actual novel questions that the new task is needed for. For instance, neither of those two works actually used bare multi-armed bandit tasks, but the casual reader might infer so from the way the paragraph is laid out.

This is a good suggestion; we agree that our original writing was ambiguous. We have followed the reviewer's recommendation and rewrote the paragraph in question (p. 3, l. 83-87):

To resolve this question, we incorporated several new key features into our experimental approach. First, inspired by two recent studies (Mancinelli et al., 2021; Zamfir & Dayan, 2022), we diverged from most previous work by employing a sensorimotor task rather than the more commonly used multi-armed bandit tasks, in which factors beyond mere choice play no role.

Furthermore, see our response to the next point for additional changes made in the discussion.

1.b The same goes for the discussion of the results in relation to these previous two studies. For example:
"A recent study on self-attribution bias revealed dynamic, reciprocal relationships between attributions and self-beliefs, supporting the attribution-self-representation cycle theory (Zamfir & Dayan, 2022). They show that participants were more likely to update their beliefs about their abilities when they attribute outcomes to themselves rather than external factors. This aligns with our findings, which show a higher error sensitivity for positive scores compared to negative scores, indicating that people tend to credit themselves for successes more readily than attribute failures to their actions"
This felt unclear to me. Related:
"These findings illustrate how erroneously attributing successes to personal skill and failures to external factors can significantly affect subsequent behavioural strategies, often resulting in future suboptimal decisions (Garrett & Daw, 2020)."
This seems a point where to discuss Mancinelli et al. -- since they show that internality is linked to learning from failure.

We agree that these points were not particularly clear, and following the reviewer’s recommendation we have rewritten the relevant paragraphs in the discussion, as well as added a more explicit reference to Mancinelli et al.

discussion, first part (p. 16, l. 506-526):

A recent study revealed dynamic, reciprocal links between outcome attributions and self-beliefs, consistent with the attribution-self-representation cycle theory (Zamfir and Dayan 2022). Participants were more likely to update their beliefs about their abilities when they attributed outcomes to themselves rather than external factors. With increasing skill estimates, participants took more credit for wins and less blame for losses. The authors also found a higher learning rate for negative than positive feedback. A different set of studies also reported greater error sensitivity following negative outcomes, driven by a self-attribution bias, as participants needed good results to earn rewards and thus learned more from failures (Dorfman et al. 2019, 2021).

In contrast, our study found greater sensitivity (α) for positive feedback. This difference likely stems from our task structure: participants had to infer the task state (random or skill) from probabilistic, implicit feedback. Negative feedback was ambiguous—arising from both motor errors in the skill condition and accurate actions in the random condition—whereas positive feedback in the random state clearly signalled a mismatch between belief and outcome. As outcomes were only partially informative of hit location, and further obscured by motor noise, participants might have weighted more diagnostic (positive) feedback more strongly. Thus, our study highlights how task structure and outcomes interact with self-attribution bias to shape error assignment and learning, which may also underlie apparently opposing results (Kim et al., 2019; Lee et al., 2024).

discussion, second part (p. 17, l. 550-556):

These findings illustrate how erroneously attributing successes to personal skill and failures to external factors can significantly affect subsequent behavioural strategies, often resulting in future suboptimal decisions (Garrett and Daw 2020). In a similar context, internality has been specifically linked to enhanced learning from failure (Mancinelli, Roiser, and Dayan 2021). There, high expectations of controllability increased learning from negative outcomes, helping subjects quickly recognize unachievable goals and protect their optimistic beliefs about control.

2.a One of the main points of the paper is to show that altered performance perception lie at the origin of people's self-attribution biases. The authors could do a slightly better job at proving this. As far as I understand, the main point is in the paragraph:
"Comparisons between the full model and alternative, simpler versions (e.g., with a single score-independent error sensitivity or constant term, true threshold, and no retention factor) confirmed that the full model better accounted for participants' choice strategies (see model comparison results in Supplementary Fig. 5). In addition, the model comparison further highlighted that the perceptual inflation (subjective threshold) played a larger role than overall positivity bias (error sensitivity and constant term) in determining behaviour, given the larger AIC difference from the full model when this parameter was fixed to the true threshold value (Supplementary Fig. 5)"
For at least the two models quoted in the last sentence, then, it would probably be good to perform model recovery in terms of the average AIC scores quoted. Either way, it would be good to point readers to exactly where it is shown that "perceptual inflation (subjective threshold) played a larger role than overall positivity bias (error sensitivity and constant term) in determining behaviour". The sentence does not reference figures or tables.

Thank you for the suggestion. As part of our model comparison analyses, we found that the fixed threshold model was worse than the symmetric model; thus, the threshold appears more critical than error sensitivity and the constant term. We clarified that Supplementary Fig. 5 suggests the importance of the subjective threshold. In addition, we have expanded on this point in the results section to better report

and highlight the full results and how they support the interpretation that altered performance perception underpins people's self-attribution bias.

results (p. 8, l. 270-281):

Our model comparison analysis further strengthened these results. We compared the full model with alternative, simpler versions. In particular, we were interested in the direct comparison with a single feedback-independent error sensitivity or constant term, true threshold, and no retention factor. The full model better accounted for participants' choice strategies (mean AIC: 463.9, all other models ΔAIC > 19, see Supplementary Fig. 5). In addition, the model comparison further highlighted that the perceptual inflation (subjective threshold θ) played a larger role than overall positivity bias (error sensitivity α and constant term β) in determining behaviour, given the larger AIC difference from the full model when this parameter was fixed to the true threshold value ("true threshold", mean AIC: 525.5) than when either error sensitivity or constant term were set to symmetric across feedback type ("common error sensitivity", mean AIC: 483.3; "common constant term", mean AIC: 502.0, Supplementary Fig. 5).

discussion (p. 15, l. 479-484):

Simulations and model comparison analyses validated this result and rejected alternative interpretations. First, the model with a fixed threshold was significantly worse than the model with the subjective threshold at explaining participants' data ($P < 0.001$, average ΔAIC = 61.6). Second, the model with a fixed threshold was also significantly worse than alternative models with feedback-independent error sensitivity ($P < 0.05$, average ΔAIC = 23.5) or constant term ($P < 0.001$, average ΔAIC = 42.2).

Finally, we followed the reviewer's suggestion and performed a model recovery analysis (see figure below). For this analysis, we first simulated data with each model using parameters within the range of estimated values from our participants' data, then fit each model to simulated data. Finally, we plotted and compared model fits using AIC. For all cases, the original ground truth model was consistently ranked as best fitting, in addition to the full model, since all alternative models are nested versions of the full model in which a parameter has been set to a fixed value. The figure below has now been added to the supplementary materials (Supplementary Fig. 9).

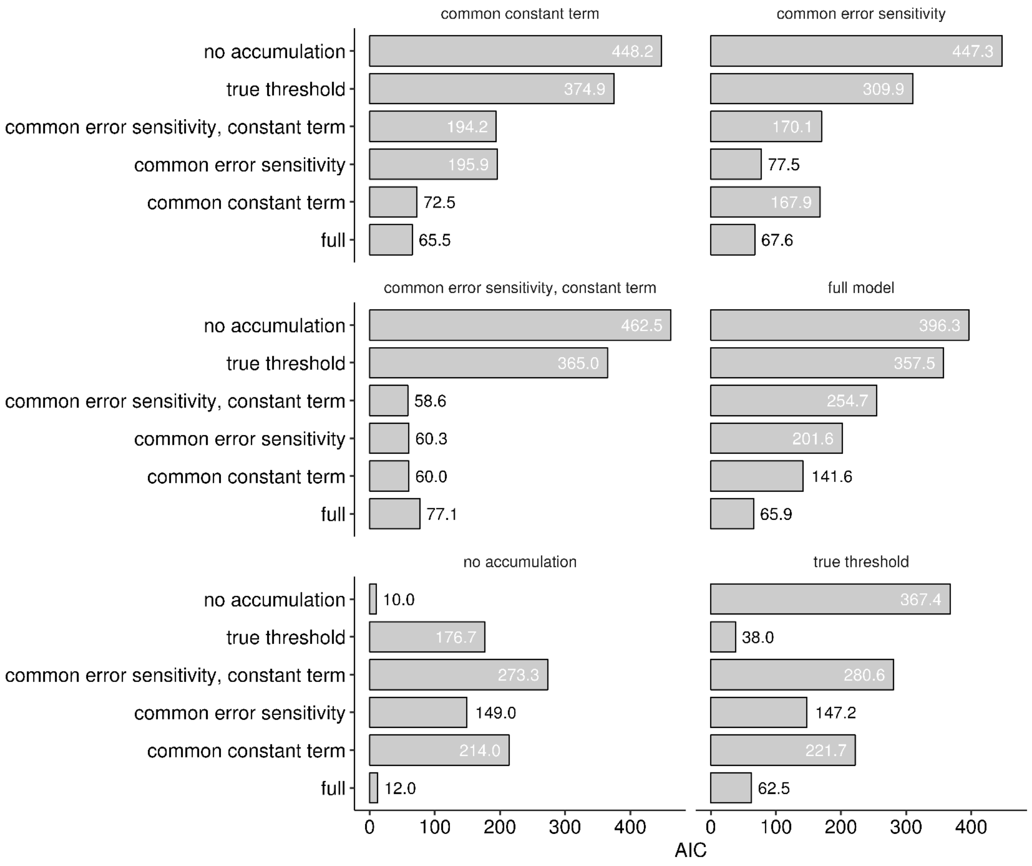

We have also added the following text in results (p. 9, l. 299-304):

Refitting simulated data with the entire set of models (full, simpler alternatives) highlighted the robustness of the model. The best fit model was the same as the underlying ground-truth, generative model (e.g., we recovered the full model when the full model had been used to generate simulated data, Supplementary Fig. 9). Together, these simulations, parameter and model recovery analysis demonstrate the robustness of the model, but also its ability to capture multiple contributing factors in participants choice behaviour and self-attribution bias.

2.b Further, the authors should comment on why models have been fit separately per subject. This should be at least touched upon since the standard nowadays is to model subjects as random effects.

We adopted a subject-wise fit instead of a hierarchical modelling approach because we could capitalise on the large amount of data per participant and were also interested in the population variance. We have now clarified this in the methods section of the manuscript.

3. The wording seems a tad unfortunate on the task state inference snippet (Figure 1, panel B). Good and skill are positively valued terms; Bad (I assume) would fit better with random. A more neutral way of putting it forward (e.g. giving the participant an altered distance from the centre, for example) could have been an alternative. Can the authors clarify this and its potential impact on results?

Thank you for the suggestion. It is true that ‘skill’ and ‘random’ might have affective connotations in certain cultures. The task was run in Japan, where this affective nuance might not apply or at least be weaker than one might expect in, for example, Western cultures. In general, in Japanese, both ‘random’ (ランダム) and ‘skill’ (スキル) are considered relatively neutral terms. We note, however, that it is possible some participants might have viewed ‘skill’ as tending towards positive affect.

4. Figure 4 appears to be chopped on the left-hand side. The Y-label is missing, and the panel letter is unreadable.

Thanks, this has been revised.
