# Supplementary material for: Blaming luck, claiming skill: Self-attribution bias in error assignment
Source: PLoS Comput Biol. 2025 Dec 16;21(12):e1013787. doi: 10.1371/journal.pcbi.1013787 (PMC12753049; doi:10.1371/journal.pcbi.1013787)
Supplement: Attachment — Submitted filename: RebuttalLetter.pdf [file pcbi.1013787.s005.pdf]

Reviewer #1

I appreciate the added nuance in the interpretation of results and discussion, and the increased clarity in the presentation of the modelling. I believe the paper is substantially better.

We thank the reviewer for their supportive stance on the revised manuscript.

I do, however, have two remaining content-related comments.

First of all, the authors refer to 2 aspects as additional evidence in favour of a perceptual contribution to the bias and against the interpretation of the theta parameter as encoding beliefs about task difficulty. These two aspects are: the drop in confidence near the inferred subjective threshold and the fact that participants who overestimated their performance in the score prediction task also had larger objective thresholds. I do not find this argument convincing, as both of these observations are, in fact, consistent with a threshold encoding beliefs about task difficulty.
Regarding the first one: if a participant believes feedback is good/bad based on a threshold chosen by the experimenter, then it is not surprising that their confidence will be lower near the value of the threshold they believe is used than far away from it.
Regarding the second one: if a participant believes the threshold for obtaining positive feedback is very stringent, it is not surprising that they will attempt to hit with higher precision than if they believe the threshold to be lax.

We agree with the reviewer that in principle these additional findings could also reflect beliefs about the experimenter's criterion. However, several features of our task and results suggest that the subjective threshold reflects, at least partly, an internal perceptual representation of motor precision, rather than only a belief about externally imposed difficulty:

(i) We here used a sensorimotor task as opposed to the more common bandits or other cognitive tasks. As such, the task intrinsically requires participants to form a perceptual representation of their own sensorimotor ability.
(ii) The true boundary is unknown to participants, and they do not know the exact location of their own mole hits. Participants have only weak access to hit locations, due to perceptual noise about their arm/hand movements and the location where they hit the screen. Thus, there is a built-in sensory/perceptual component, which should be reflected in the subjective threshold.
(iii) The U-shaped confidence pattern tracks the subjective threshold. Considering the above arguments (i) and (ii), confidence would reflect belief about task difficulty only if participants had access to exact hit location information (which they did not).
(iv) If the subjective threshold mainly reflected belief about task difficulty, we would expect average confidence to track the subjective threshold across participants. I.e., participants who thought the task had a liberal criterion would also be more confident about their choices. This was not the case ($r = -0.0028$, $p = 0.98$), suggesting the subjective threshold captures more than belief about task difficulty, such as perceptual effects.

(v) Participants with poor motor performance (hits far from centre) consistently overestimated their ability (i.e., in the initial score estimation task). Participants had no prior knowledge of the threshold at this stage of the task, and the distribution of these hits determined the true threshold in the main inference task. Regarding this point (second aspect pointed out by the reviewer above), we feel there may have been a misunderstanding. See also our response to the second comment below for further details on this result and its interpretation.

In summary, we acknowledge that perceived task difficulty and self-estimated ability are conceptually intertwined and difficult to tease apart. Based on the arguments and assumptions developed above, we find the data to support a primarily perceptual interpretation: θ captures the internal sensory calibration of one's own motor accuracy, rather than an inferred external rule. The confidence pattern and cross-task correlation are therefore best understood as expressions of a distorted self-percept of performance, rather than as beliefs about the experimenter's criterion.

To better capture these nuances and the important points raised by the reviewer, we have modified the manuscript as follows: in the discussion, we present both views, stating that we favour the perceptual interpretation but highlighting that the data, at present, are not conclusive. We hope the reviewer will be satisfied with this more nuanced framing as well as more clearly described results and interpretations. See below for changes made in the manuscript:

Discussion, ll. 595-625:

> Ultimately, while we interpret the subjective threshold parameter $\boldsymbol{\theta}$ as reflecting participants' internal estimate of their own motor precision, we acknowledge that it can also be viewed as encoding beliefs about task difficulty—i.e., participants had precise perception of where they hit, but they had imprecise inference of the boundary between good and bad performance. Teasing apart perceived ability from inferred task demands is inherently challenging, as both likely contribute to the setting of $\boldsymbol{\theta}$. However, we suggest that the interpretation as an expression of the task difficulty appears less plausible in our design. Specifically, participants were trained during a practice session (80 trials) with explicit feedback on the true state, which should have allowed them to learn the overall difficulty and structure of the task. We therefore propose that $\boldsymbol{\theta}$ may primarily capture the remaining uncertainty: participants' perceptual uncertainty about their own motor performance. The nature of our task, which combines inference with sensory-motor control, inherently introduces such uncertainty, preventing perfect access to the actions performed. Our data is consistent with this interpretation. We found that participants who overestimated their motor accuracy in the initial score prediction task also exhibited larger objective thresholds, indicating lower accuracy in hitting the centre, during the main task (Supplementary Fig. 18). This finding underscores the positive correlation between motor ability and bias in perception of motor ability.
>
> Note that we also found a modest underestimation in high-performing individuals. This pattern is consistent with established psychophysical effects, in which individuals tend to be overconfident about their successes when they perform poorly (Lichtenstein,

Fischhoff, and Phillips 1982; Zylberberg, Roelfsema, and Sigman 2014). In contrast, higher perceptual ability can lead to more conservative confidence bias placement (Fleming and Lau 2014), and increased signal strength can reduce overconfidence by tightening confidence thresholds (Shekhar and Rahnev 2021), potentially resulting in a modest underestimation of their performance. Our findings replicated this systematic skew in perception, and it is reasonable to infer that the same perceptual skew was present during the main task and affected participants' rule inference. However, this evidence remains indirect. We cannot entirely rule out the possibility that the threshold parameter also encodes cognitive evaluation of the task difficulty to hit the centre. Further research, including well-designed experiments, is required to explicitly decouple these two factors.

Results, ll 372-373:

Confidence was lowest near the boundary, and highest at the extreme (centre, or edges). This result supports the validity of the θ parameter as a subjective threshold.

Second, the way the authors describe and interpret Supplementary figure 18, which they describe in the discussion (lines 590-591) as "underscoring the positive correlation between motor ability and perception of motor ability" seems slightly inaccurate. A positive correlation between motor ability and perception of motor ability would mean that a lower threshold(i.e. higher accuracy in motor performance) was associated with a smaller absolute deviation between predicted and actual score (i.e higher accuracy in perception of ability). This is slightly different from what the plot shows. The plot does show that a higher objective threshold (therefore lower motor accuracy) is associated with a more positive score prediction error. However, lower objective thresholds seem (plotting the absolute prediction error on the y axis would indicate whether this is indeed the case or not) to correspond to more negative, rather than smaller absolute value prediction error. This is not necessarily consistent with the way the figure is described. It is also not clear why high motor accuracy would be associated with underestimation of score, under the perceptual effect that the authors are arguing for.

We thank the reviewer for the close reading. In hindsight, the phrasing in the manuscript was confusing, and we have now sought to clarify what Supplementary Fig. 18 was intended to show. The figure was not designed to assess the accuracy of self-evaluation (which would require absolute prediction errors, as the reviewer rightfully noted), but rather the directional bias in participants' estimation of their motor performance.

We modified the caption of Supplementary Figure 18 so that readers can better understand that the y-axis represents participants' overestimates/underestimates of their motor ability:

A larger threshold means participants hit, on average, further from the centre of the mole, and a positive error means participants overestimated their ability to hit the centre. Thus, the x-axis represents motor ability, while the y-axis represents bias in the subjective evaluation of motor ability.

We acknowledge that this figure only provides indirect evidence for a perceptual effect. However, this figure does indicate the presence of a skewed perception of hits, and it is reasonable to assume that this effect would impact behaviour in the main inference task. Additionally, if participants did not have a skewed/inflated perception, they would learn the task difficulty well during the practice with full feedback.

Thus, we explicitly wrote our interpretation of the figure in the discussion more clearly:

ll. 609-612

> We found that participants who overestimated their motor accuracy in the initial score prediction task also exhibited larger objective thresholds, indicating lower accuracy in hitting the centre, during the main task (Supplementary Fig. 18). This finding underscores the positive correlation between motor ability and bias in perception of motor ability.

Finally, considering the reviewer's last point on why high motor accuracy would be associated with underestimation of score, we share the same view that this is an interesting asymmetry. One plausible explanation is that high-precision participants set stricter internal criteria and thus discounted near-perfect hits, resulting in a conservative bias. In contrast, lower-precision participants used broader internal criteria and inflated their scores, leading to a liberal bias. This pattern is consistent with established psychophysical effects, in which individuals who perform poorly tend to be overconfident about their successes (e.g., Lichtenstein et al., 1982; Zylberberg et al. 2014). In contrast, higher perceptual ability can lead to more conservative confidence bias placement (Fleming and Lau, 2014). Increased signal strength can reduce overconfidence by tightening confidence thresholds (Shekhar & Rahnev, 2020), potentially resulting in a modest underestimation of performance.

We have added this and concluded with a note of caution in the relevant paragraph, see our response to the first comment above with a text excerpt from the discussion.

Lower level: the phrase starting with "the task was calibrated.." on lines 135-139 is difficult to parse and contains typos.

The sentence has been corrected. See below for the new version:

> The task was calibrated such that participants would obtain positive feedback with similar probabilities in both 'random' and 'skill' task states. This was achieved by setting the threshold for the positive feedback in the skill state individually as the median distance of each participant's hit location distribution during the preliminary score prediction task.

Reviewer #2

I thank the authors for their meticulous responses, which greatly clarified my earlier concerns. I had initially misinterpreted the type of self-attribution bias they aimed to capture, thinking they sought to document this bias even when participants had accurate perceptions of their abilities. This had made their design difficult for me to follow. With the revised version, however, the theoretical story is much clearer. I also appreciate the extensive robustness checks and model recovery analyses, which are highly reassuring to me. Overall, they addressed my main concerns, and I recommend acceptance based on the new version.

We thank the reviewer for their careful consideration of the revised manuscript and endorsement of this work.

The only remaining comment concerns Supplementary Figure 2. From my understanding, the authors conducted a two-sided Wilcoxon signed-rank test with the null hypothesis of no systematic difference between the two states, and reported a p-value of 0.01. By conventional interpretation, this would indicate a statistically significant difference, whereas the figure legend states that “the ratio of positive scores was relatively similar across states.” I may be missing some nuance here, but I would appreciate clarification of how these statements are reconciled.

The wording of the result reported in Supplementary Figure 2 was indeed confusing, and we thank the reviewer for bringing this to our attention. We have now amended the legend and main text as follows:

> The task was calibrated such that participants would obtain roughly 50% positive scores in both skill and random states. Note that, nevertheless, the overall ratio of positive scores was significantly larger in the skill state compared to the random state at the group level (Wilcoxon signed-rank test, $Z = 2.58$, $p = 0.01$).

Reviewer #3

I'm satisfied with the responses from the authors.

We thank the reviewer for their positive take on our revised manuscript.
