# Supplementary material for: Blaming luck, claiming skill: Self-attribution bias in error assignment
Source: PLoS Comput Biol. 2025 Dec 16;21(12):e1013787. doi: 10.1371/journal.pcbi.1013787 (PMC12753049; doi:10.1371/journal.pcbi.1013787)
Supplement: Attachment — Submitted filename: Reviewer Comment.pdf [file pcbi.1013787.s002.pdf]

Comments for “Blaming Luck, Claiming Skill: Self-Attribution Bias in Error Assignment”

I commend the authors for their innovative approach to investigating self-attribution bias. Through a carefully designed experiment, they tried to not only document the existence of this bias but also propose and validate an underlying mechanism driving it. I appreciate the authors' effort to deviate from conventional experimental designs and their thorough modeling analyses. However, I have several concerns regarding the task design, the potential influence of confounding factors, and the validity and robustness of their modeling analyses.

Data Filtering:

I am concerned about the authors’ decision to pool data from all confidence levels without differentiating between them. Initially, I believed that the confidence feature was intended as a filter, allowing responses with low confidence (levels 1 and 2) to be excluded from analysis. While the authors separately discuss the confidence data in their results, pooling choices from low-confidence trials (basically guesswork) with high-confidence choices is problematic. The analysis would benefit from excluding low-confidence data and reconsidering the way these responses are treated in the regressions, tests, and modelling analyses.

Task Design:

The primary observed effect of self-attribution bias is that participants are more likely to attribute their performance to “skill” when receiving a positive score. However, it remains unclear whether self-attribution bias is the only or primary driver of this effect.

A key assumption underlying the authors' conclusions is that participants perceive a 50% chance of success in “skill” sessions, which is the same as in “random” sessions. However, I am skeptical that this condition is met, and the observed effect might be driven by inference bias, prior beliefs, and psychological accounts:

- **Inference Bias**: Participants have no idea of the threshold and have to infer it. Given the way they design the task, I would be truly surprised if participants accurately inferred the threshold. Naturally, the observed effect could be because they inferred the threshold to be larger than the true value, so the probability of winning is >50% in “skill” sessions, but this may not necessary be due to self-attribution but simply some inference biases. I am wondering if the overestimation of the threshold can be explained by some well-documented inference biases. If so, what they observed is an existing inference bias which manifests as “overconfidence” in this task.

- **Prior Beliefs from Training:** The task is cognitively demanding: they must actively infer in each trial exactly where they just hit and where is the threshold. It is likely that they will use more simplified approaches such as forming a belief about their probability of winning in the skill state and using this belief to make inferences. In this case, the training phase, where participants are exposed to customized thresholds, could contribute to the formation of optimistic prior beliefs.
    - If many participants achieve greater than 50% success during training, they may carry this belief into the main task, resulting in the attribution of "skill" following positive feedback[1].
    - The authors attempt to justify this possibility with Supplementary Figure 2, but I find the graph unconvincing:
        - The upward skew and large volatility observed in the data suggest that an optimistic prior belief is a reasonable assumption for a majority of the participants.
        - Such post-game stats are not relevant to participant belief. We don't know how and if participants updated their belief or what beliefs they had.
        - Supplementary Figure 2 legends: "*Wilcoxon signed-rank test, Z = 2.58,* ***p = 0.01***". The authors should have conducted a two-sided test here. If the conclusion is the scores are similar across states (they fail to reject the null hypothesis), shouldn't the p-value be close to 1 instead of 0?
- **Ease of Performance Evaluation:** The graphical design of the task may unintentionally bias participants. It appears easier for participants to evaluate very good motor performance than very poor performance[2]. Note that this is different from what the authors discussed on page 5: not the positive scores are easier to evaluate, but good motor performance is easier to recognize. This difference in ease of evaluation could explain the observed effect. To examine this, the authors could consider filtering the data to focus on only trials with high confidence (level>=3) and unambiguous motor performance[3], and examine whether the observed effect is because there are more trials where participants performed unambiguously well. If yes, the observed effect is due to the task design and psychological accounts instead of self-attribution bias. Otherwise, if the "low performance" group dominates and we still observe the effects among the filtered data, it would be a more compelling evidence of self-attribution bias.

---

[1] Even if they didn't perform well enough in training, they may believe that they have improved from the practices and thus be optimistic.
[2] It is intuitive to tell whether I hit closer to the centre point (a small dense area) than outside the threshold (large circular area)
[3] The hit is either very close the centre or very far from the threshold.

- **Incentives and Asymmetric Effort**: The bonus feature, which rewards participants for correctly estimating the task state and achieving a positive score, could also introduce bias. Since no reward is given for correctly estimating the state with a negative score, participants may exert less effort during these trials, skewing the results in favor of the "skill" state.
- **Performance Volatility and Sample Size Justification**: The volatility of participants' performance raises concerns about the sample size and the power of the analyses. Without justification of the sample size or a power analysis, the robustness of the results is uncertain. Furthermore, I presume that no pre-registration was conducted.

Modeling Setup:

- I was surprised to see that the prediction error in the model was treated as binary. The assumption that participants clearly know whether they hit inside or outside the threshold is unrealistic, given that the participants may not have actively inferred the threshold and their hit location. Moreover, the accuracy of the hit (i.e., how close it is to the threshold; dt-theta in the models) should matter. For example, a hit near the threshold would feel ambiguous to the participant, and the prediction error would likely be smaller regardless of the results. The model's treatment of prediction error as binary may not accurately reflect the nuances of cognition.
- **Incorporating Confidence into the Model**: While the authors tried to justify this, the model does not appear to incorporate confidence levels into the decision-making process or the model fitting. Ideally, confidence could be generated jointly with the choice data, reflecting the participant's certainty about their performance. This might be achieved by incorporating the accuracy of the hit; see above. If such modelling is really intractable, the least thing to do is incorporating the confidence data into model fitting, e.g., if the confidence in each trial is high, more weight should be given to the LL in that trial.

Modeling Analyses:

- **Parameter Recovery**: Many of the analyses rely on fitted parameters, which are known to be prone to uncertainty. While the authors provide some parameter recovery (Supplementary Figure 8), this does not fully validate their methodology. The correlation between parameters alone cannot justify the parameter recovery.
    - A more rigorous validation would involve conducting simulations to assess the likelihood of false positives and false negatives. For instance, one could simulate choices without bias (e.g., assuming no difference in alpha between positive and negative feedback) and then fit the model to

see if the results lead to false positives (detection of a higher alpha for positive feedback). Similarly, simulations with varying levels of bias could assess the model's ability to detect biases without incurring significant false negatives. More professional robustness checks would involve varying the strength of the simulated biases and to assume that only a certain portion of agents is subject to the bias.

- **Model Comparison**: Instead of relying solely on AIC and BIC criteria for model comparison, I suggest that the authors use cross-validation. This method would involve fitting the model to the first half of each participant's choices and then testing the model's ability to predict the second half. The performance of the models could then be compared based on out-of-sample likelihood. If the authors really want to keep the current comparison criteria, I recommend conducting a more thorough recovery analysis for all model variants: simulating the choices from one model variant and run model comparison, the true variant should outperform all alternatives.

Minor Comments:

- On page 5, to test the effect of the score, it is interesting to see whether the results are robust using the 100-point score instead of binary results. Participants in the game not only see the binary results but may also have a rough feeling about the accuracy of the hit, c.f., my previous comments.
- On page 6: "*Interestingly, we found that where participants hit the moles in relation to the centre differed depending on the objective task state (see Supplementary Fig. 4A-B, hits were closer to the centre in the skill state than the random state).*" I thought this is not surprising. If their effort did not matter in the random state, of course the motor performance is worse. Not sure if I missed other interesting insights here.
- On page 6: "*Together, these results suggest that participants dynamically adjusted their hit location. However, there was no evidence that they did so strategically, in which case we should have seen an effect only in the 'skill' state and not in both, as reported here.*" I don't understand the logic here which seems to assume that participants can perfectly infer the underlying state. Participants didn't know the true state and there could be significant lag effects in their inferences, so of course they would still implement their strategy in the random state.
- The exclusion criteria should also account for participants with consistently low confidence (e.g., those with confidence level <3 for a large proportion of trials), as their data quality in in doubt.

- Page 27: "*Finally, we calculated Spearman's rank correlation coefficient for each parameter and generated the corresponding confusion matrix (Supplementary **Figure 7**).*" A typo, should be Figure 8.
